# Facilitated Variation: How Evolution Learns from Past Environments To Generalize to New Environments

**DOI:** 10.1371/journal.pcbi.1000206

**Published:** 2008-11-07

**Authors:** Merav Parter, Nadav Kashtan, Uri Alon

**Affiliations:** Department of Molecular Cell Biology, Weizmann Institute of Science, Rehovot, Israel; Washington University, United States of America

## Abstract

One of the striking features of evolution is the appearance of novel structures in organisms. Recently, Kirschner and Gerhart have integrated discoveries in evolution, genetics, and developmental biology to form a theory of facilitated variation (FV). The key observation is that organisms are designed such that random genetic changes are channeled in phenotypic directions that are potentially useful. An open question is how FV spontaneously emerges during evolution. Here, we address this by means of computer simulations of two well-studied model systems, logic circuits and RNA secondary structure. We find that evolution of FV is enhanced in environments that change from time to time in a systematic way: the varying environments are made of the same set of subgoals but in different combinations. We find that organisms that evolve under such varying goals not only remember their history but also generalize to future environments, exhibiting high adaptability to novel goals. Rapid adaptation is seen to goals composed of the same subgoals in novel combinations, and to goals where one of the subgoals was never seen in the history of the organism. The mechanisms for such enhanced generation of novelty (generalization) are analyzed, as is the way that organisms store information in their genomes about their past environments. Elements of facilitated variation theory, such as weak regulatory linkage, modularity, and reduced pleiotropy of mutations, evolve spontaneously under these conditions. Thus, environments that change in a systematic, modular fashion seem to promote facilitated variation and allow evolution to generalize to novel conditions.

## Author Summary

One of the striking features of evolution is the appearance of novel structures in organisms. The origin of the ability to generate novelty is one of the main mysteries in evolutionary theory. The molecular mechanisms that enhance the evolution of novelty were recently integrated by Kirschner and Gerhart in their theory of facilitated variation. This theory suggests that organisms have a design that makes it more likely that random genetic changes will result in organisms with novel shapes that can survive. Here we demonstrate how facilitated variation can arise in computer simulations of evolution. We propose a quantitative approach for studying facilitated variation in computational model systems. We find that the evolution of facilitated variation is enhanced in environments that change from time to time in a systematic way: the varying environments are made of the same set of subgoals, but in different combinations. Under such varying conditions, the simulated organisms store information about past environments in their genome, and develop a special modular design that can readily generate novel modules.

## Introduction

The origin of the ability to generate novelty is one of the main mysteries in evolution. Pioneers of evolutionary theory, including Baldwin [1], Simpson [2], and Waddington [3],[4], suggested how useful novelty might be enhanced by physiological adaptations and by the robustness of the developmental process. These early theories were limited by a lack of knowledge of the molecular mechanisms of development.

Recent decades saw breakthroughs in the depth of understanding of molecular and developmental biology. Many of these findings were unified in the theory of facilitated variation [5], presented by Kirschner and Gerhart, that addresses the following question: how can small, random genetic changes be converted into complex useful innovations? In order to understand novelty in evolution, Kirschner and Gerhart integrated observations on molecular mechanisms to show how the current design of an organism helps to determine the nature and the degree of future variation. The key observation is that the organism, by its intrinsic construction, biases both the type and the amount of its phenotypic variation in response to random genetic mutation [3], [4], [6]–[10]. In other words, the organism seems to be built in such a way that small genetic mutations have a high chance of yielding a large phenotypic payoff.

To understand FV, it is important to compare it to the related concept of evolvability. A biological system is evolvable if it can readily acquire novel functions through genetic changes that help the organism survive and reproduce in future environments [11]. Evolvability is composed of two aspects: 1) variability: the capacity to generate new phenotypes 2) fitness: the fitness of the new phenotypes in future environments. Most studies of evolvability focused on the first aspect, variability. Such studies measured the range and diversity of the phenotypic variation that can be generated by a given mutation, usually without discerning between potentially useful phenotypes and non-useful ones [12]–[16] (for an interesting exception see Ciliberti et al [17]). FV theory adds to previous considerations by focusing on the nature of the generated variation, and specifically on the organism's ability to generate novel phenotypes which are potentially *useful*.

**Figure 1:**
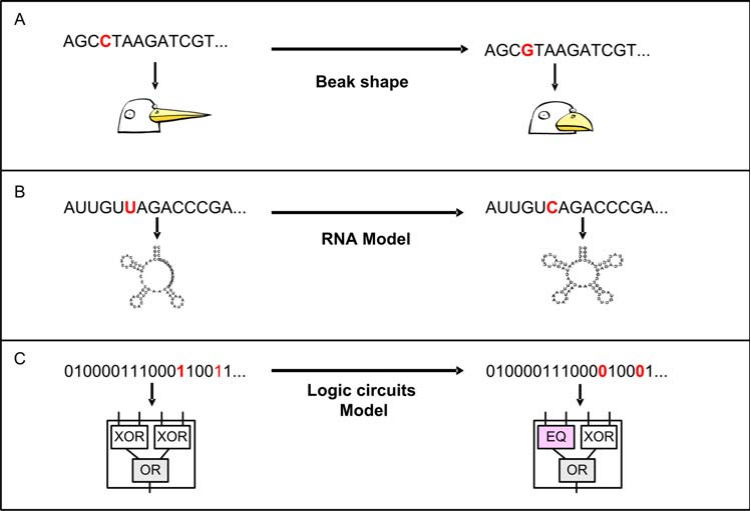
A small number of mutations evokes large useful phenotypic adaptation in systems showing facilitated variation. (A) Beaks of Darwin's finches. (B) RNA secondary structure evolved under modularly varying goals (MVG). (C) Logic circuit evolved under MVG of decomposable Boolean functions.

Facilitated variation (FV) is made possible by certain features of biological design. One of these is the existence of ‘weak regulatory linkage’ [5],[10],[18], where general and non-instructive signals can trigger large pre-prepared responses. For example, changes in growth hormone concentration at a localized position (limb bud in an embryo) can trigger large useful changes in the shape of the limb, driven by the conserved mechanisms for growth of bones, muscles, blood vessels, and nerves [19]. A good example is the ease of changing beak shapes with any of many possible mutations that affect the concentration of a single morphogenic factor [20] (Figure 1A). In weak regulatory linkage, the information about the output is pre-built into the regulated system without instruction from the regulator, which only selects between states. Such regulatory organization reduces the constraints for evolving new regulations and for generating complex potentially useful phenotypes.

An additional feature that is important for FV is modular design [21]–[24], seen for example, in the highly conserved body-plan of the embryo [25],[26] and in the compartmental organization of gene regulation and signaling networks [27]. Modularity helps to relieve the concern that a mutation might interfere with many different parts of the organism. With properly designed modularity, variation within each module can be generated without harming other modules [28]–[31].

Facilitated variation can be in principle studied experimentally, for example by generating mutants and scanning the types of phenotypes generated. For example, a study on mutants of the *lac* regulatory region indicated that the shape of the gene input function is channeled in directions of AND-like and OR-like functions, rather than other possibilities [32].

An open question is how does FV spontaneously evolve? It is not clear how selection in a present environment can lead to designs that increase the probability of useful changes in future environments. How does evolutionary theory account for the emergence of special designs that make it easy to generate novel and useful variation?

The key point in our study is the observation that environments in nature do not vary randomly, but rather seem to have common rules or regularities [33]–[35]. Specifically, environmental goals faced by organisms or molecules may be thought of as composed of a combination of subgoals [33]. When environments change, the organisms encounter a new goal that is still made of the same or similar subgoals. For example, on the level of the organism, the same subgoals, such as digesting food, avoiding predation, and reproducing, must be fulfilled in each new environment but with different nuances and combinations. On the level of cells, the same subgoals such as adhesion and signaling must be fulfilled in each tissue type but with different input and output signals. On the level of proteins, the same subgoals, such as enzymatic activity, binding to other proteins, regulatory input domains, etc., are shared by many proteins but with different combinations in each case.

One may thus propose that in many cases, the different possible environments share a language of modularity, in the sense that they are all made of certain combinations of a set of subgoals. We thus test the possibility that under such patterned varying environments, the organism can learn over many generations the language common to the environments encountered in its past. We ask whether FV arises in such systematically varying environments, by measuring the ability of simple model systems to adapt to new, previously unseen goals, which are in the same language as past goals.

We employ two well-studied model systems: combinatorial logic circuits [33],[34] and RNA secondary structure [12]. We find that the standard experiment of setting a goal which remains constant over time leads to highly optimized systems that show little FV. In contrast, FV is readily generated under modularly varying goals (MVG), in which goals change over time but share the same subgoals [33]. We find that MVG evolution enhances the ability to generate novel phenotypes as long as novelty is modular: phenotypes with novel modules or novel combinations of modules. We show that organisms under MVG store information about past goals in their genomes, and evolve weak linkage that allows small genetic changes to unleash large phenotypic responses that do not ruin the modular structure of the organism. Our study thus suggests that environments that change in a systematic fashion promote the evolution of facilitated variation, and leave an imprint on the evolvability properties of the organisms, allowing them to generalize to new conditions that are in the same language as past conditions.

## Results

### Description of the Model Systems

#### Combinatorial logic circuit model

The first model system in this study is circuits made of logic gates, evolved toward a desired Boolean function *G*. The circuits are composed of NAND gates (NOT-AND function), have several input ports and a single output port. The fitness of the circuit is the fraction of times it computes the desired output, *G*, when evaluated over all possible combinations of the Boolean values of the inputs. The wiring of the gates is coded in a genome (string of bits). Starting with a population of random genomes, mutations are made and high fitness individuals are selected by means of a standard genetic algorithm (see Methods). The present results hold both in the presence and absence of recombination.

**Figure 2:**
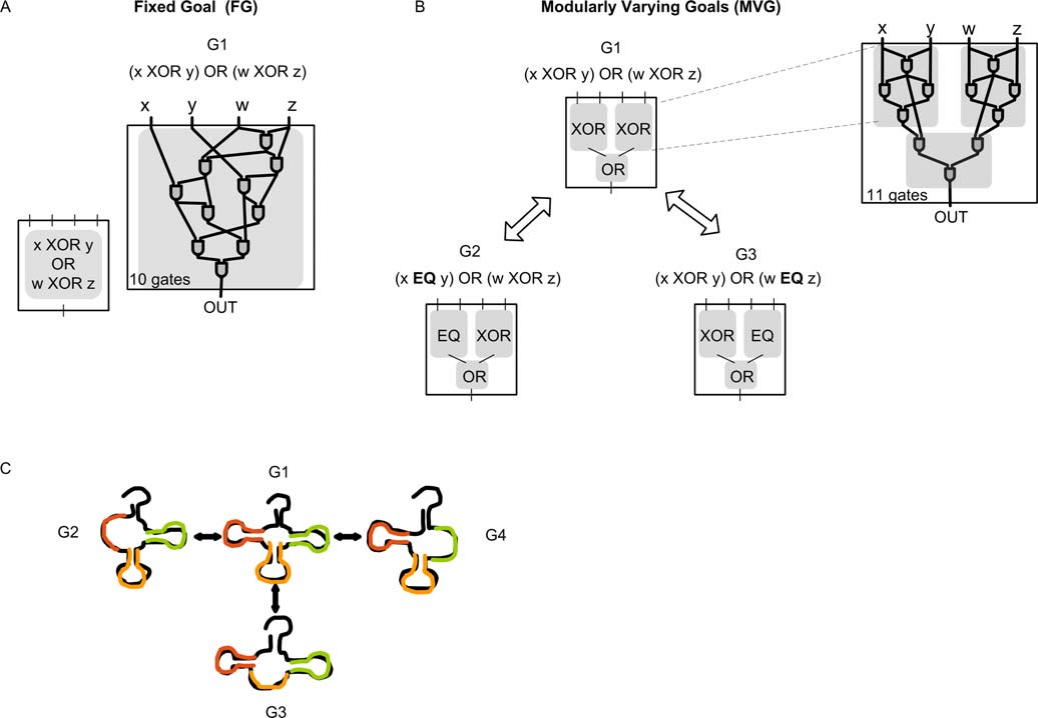
Schematic view of evolutionary goals and phenotypes in the two model systems. (A–B) Logic circuit model. (A) A typical circuit evolved by fixed-goal evolution toward goal G1. Each gate in the circuit represents a NAND gate. (B) Modular circuit evolved under modularly varying goal evolution with goals G1-G3. The circuit is composed of two XOR modules that input into a third module which implements the OR function. Each goal has four inputs and one output of the form G(x,y,w,z) = *f*(*g*(x,y),*h*(w,z)). During evolution, goal switches over time in probabilistic manner as a random walk on the graph in this figure; Note that in every switch, a single XOR module is changed to EQ, and vice versa. (C) RNA model, goal G1 is the secondary structure of a natural tRNA, goals G2, G3 and G4 are modular variants of G1, in which one hairpin loop is replaced by an open loop. The goal switches during evolution in a probabilistic manner as a random walk on the graph in the figure. Note that in every switch, a single hairpin is changed to an open loop and vice-versa.

We compared evolution of circuits under a goal that is constant over time (called here fixed-goal or FG) to circuits evolved under goals which change from time to time in modular fashion (called modularly varying goals, denoted MVG). In FG evolution, the goal is a Boolean function such as
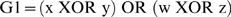
(1)where XOR is the exclusive-or function. The resulting circuits have a non-modular design, as previously found [33]. The structure is non-modular despite the fact that the goals, such as G1, can be decomposed into subgoals (two XORs and one OR operations) (Figure 2A).

In contrast, under MVG, instead of keeping the goal fixed, we switched the goal every *E* = 20 generations. These are rapid changes in comparison to the length of the simulations, 10^5^ generations. A wide range of switching times E gives similar results.

Importantly, all goals presented along MVG evolution shared the same subgoals but in different combinations (Figure 2B). For example, we evolved the circuits toward G1 for 20 generations and then switched the goal to a similar function G2, in which one of the XORs is replaced by an EQ (the EQUAL function).
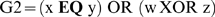
(2)and then back to G1 and so on. Similar findings were obtained with three goals, with probabilistic transitions between G1, G2 and a third modularly related goal:
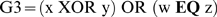
(3)Similar findings are also found when OR is changed to AND, for example G2 = (x XOR y) **AND** (w XOR z). The specific examples were chosen because XOR and EQ are the most difficult two-input Boolean functions to implement with NAND-gate circuits. Contrary to FG evolution, the circuits evolved under MVG are found to have a modular structure: they display a structural module for each of the computational subgoals [33] (e.g. two modules that rapidly rewire by mutations to serve as a XOR or EQ according to the present goal, and a third module that performs an OR operation) (Figure 2B).

#### RNA secondary structure model

In addition to logic circuits, we studied RNA secondary structures. Here, genomes are RNA nucleotide sequences, and the goal is given by a desired secondary structure. A standard RNA folding algorithm was used to determine the secondary structure of each genome sequence [36]. Fitness was based on the most stable shape (minimum free energy, denoted MFE) corresponding to the genome sequence [12]. The fitness of the sequence is then defined as 1-*d/B*, where *d* is the structural distance to the goal and *B* is the length of the sequence [34].

We evolved an initially random population of RNA sequences toward predefined secondary structure using a standard genetic algorithm. We present in detail the example of a ‘clover leaf’ tRNA structure [12], but other structures gave similar conclusions, see Text S1 section 1.2. This clover leaf has three structural modules, two hairpin loops and one hairpin loop with a bulge. In FG simulations, the goal remained constant along evolution. In the MVG scenario, we switched between goals in a modular way in the sense that the different goal structures shared the same library of structural modules (such as hairpin loops and open loops) but in different combinations (Figure 2C) [34].

### MVG Genotypes Adapt Rapidly When Goals Change

In the following, we mainly focus on two representative problems, logic circuits evolved towards combinations of XOR and EQ goals, and RNA molecules evolved towards cloverleaf-like RNA structure. Similar conclusions were found for all six Boolean goals studied and five other RNA structures tested, as detailed in Text S1 sections 1.1 and 1.2.

Under MVG evolution, the evolving circuits or RNA molecules were exposed to a series of goals that are related to each other by their shared set of subgoals. We find that within a few thousand generations, genomes evolve that are able to adapt rapidly, often within a single generation, to each new goal (Figure 1B and 1C). Despite the fact that the phenotypic adaptation is large (e.g. an entire hairpin changes to an unstructured open loop, or a change in about half the bits in the truth table of a circuit goal, see Methods), the adaptation is associated with a very small genetic change, usually only 1–2 mutations.

**Figure 3:**
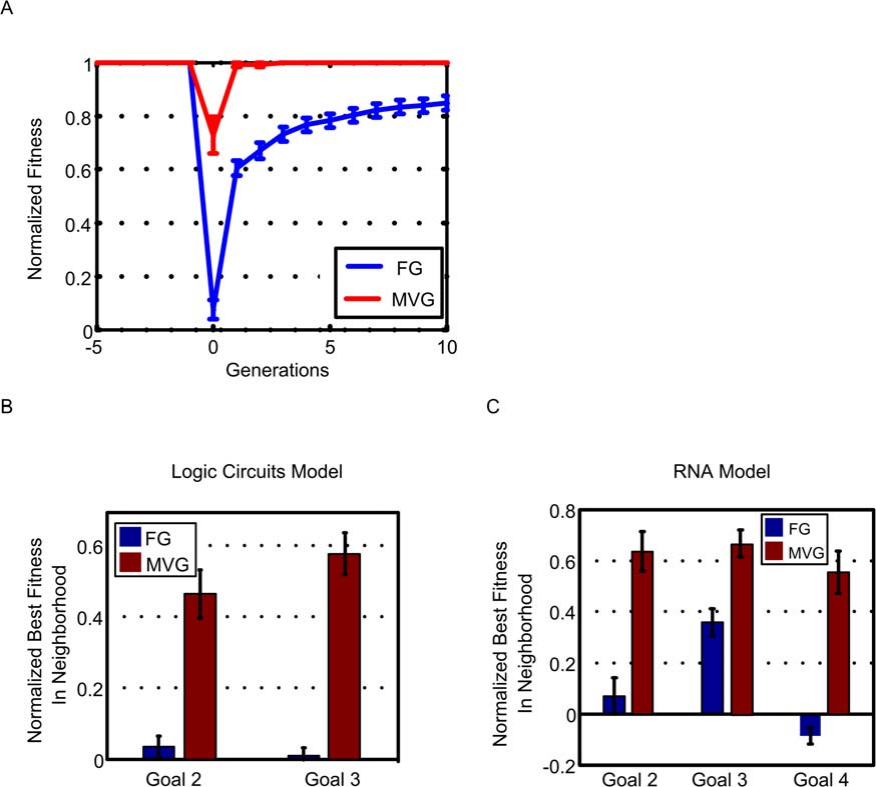
MVG-evolved organisms adapt faster than fixed-goal organisms when goals change. (A) Adaptation following a goal switch (logic circuit model). The x-axis denotes generations, where zero is the point where the goal changes to a new goal (a previously seen goal in the case of MVG). Maximal normalized fitness in the population at each time point (mean±SE) is shown. Initial populations are FG-populations evolved toward G1 and MVG-populations taken from the end of the last G1-epoch. The new goals were G2 = (x EQ y) OR (w XOR z) and G3 = (x XOR y) OR (w EQ z). Data are from 30 simulations for each scenario. (B) Maximal normalized fitness (mean±SE) for past goal G≠G1 in the genetic neighborhood of evolved logic circuits. (C) Same as in (B) but for evolved RNA genomes. The genetic neighborhood is defined as the set of all genomes different in one position from the wild type genomes.

In contrast, adaptation of organisms evolved under FG is slow when the goal is suddenly switched, even if the switch is to a goal with the same subgoals as the previous goal. FG-organisms take a dozen times more generations to satisfy the new goal (Figure 3A), and require about five times more mutations on average, than organisms evolved under MVG. The same is true for the other goals tested in Text S1. Thus, the response to changing goals is significantly slower than the response of MVG-evolved organisms to previously seen goals (Figure 3A).

### High-Fitness Phenotypes for Past Goals Are Found within MVG Phenotypic Neighborhood

We next asked what is special about the design of MVG-evolved organisms that facilitates their response to changing goals? For this purpose, we considered the phenotypic neighborhood [37]–[39], defined as the set of phenotypes that are accessible from a given genotype by a single point mutation.

We find that the phenotypic neighborhood of MVG-evolved genomes includes phenotypes that have high fitness to the past goals seen in their history (Figure 3B). This indicates that the evolved organism effectively remembers its past goals by storing information about it in its genome. In contrast, in genomes evolved under constant conditions (FG), the fitness of the neighborhood for new goals is significantly lower.

FG populations are known to evolve toward the center of the neutral network, defined as the set of all genotypes with the same phenotype that are connected by neutral mutations [40]–[42]. Thus the FG organisms are more robust to genetic mutations and their phenotypic neighborhood exhibits a lower degree of variation than the MVG organisms. These features are also found in the present study (Text S1, section 4.1). In contrast, MVG organisms seem to be located at the edge of the neutral network that is closest to the neutral networks of the previously seen goals. This implies that temporally varying environments push populations towards special regions of the neutral network.

In addition to genetic mutations, one can also study thermal fluctuations that give rise to alternative structures encoded by a single genotype [12]. Thus, in the RNA model, we considered in addition to the genetic neighborhood also the thermodynamic neighborhood: the set of structures for a given genome that have a free energy that is within 5kT of the minimal free energy (MFE state) and are therefore accessible with a non-negligible probability by thermal fluctuations [43]. We find that the thermodynamic neighborhoods of MVG-evolved genomes include structures that have high fitness for previously seen goals. The FG-evolved genomes we have tested have a thermal neighborhood whose fitness for new goals is significantly lower. In this respect, the thermodynamic neighborhood is similar to the genetic neighborhood (Figure 3C, and Text S1 section 5.2), a phenomenon called ‘plastogenetic congruence’ [12] (Text S1 section 4.1).

### The Adaptation to Previously Seen Goals Is Facilitated by Genetic Triggers

We find that the rapid adaptation to previously seen goals in MVG organisms is facilitated by key positions in the genome that can stabilize a desired sub-structure or module among other potential outcomes. We term these positions ‘genetic triggers’, since they can trigger a large and prepared phenotypic response.

**Figure 4:**
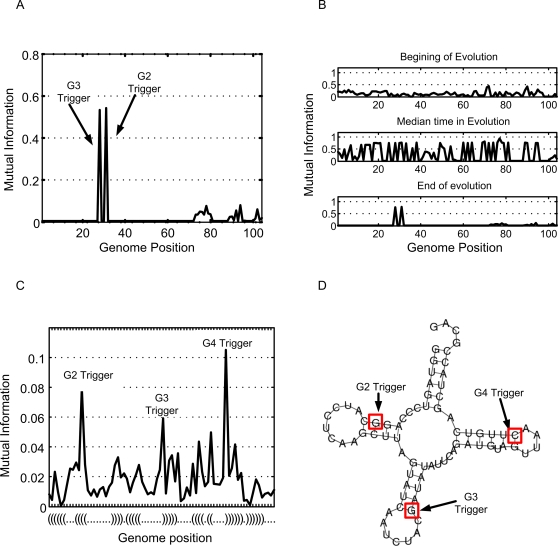
Evolution of genetic triggers. (A) Mutual information (y-axis) between the environment (goal) and the genomic content at each position of an evolved MVG circuit (x-axis). Two positions (positions 28 and 31) have high mutual information and are defined as genetic triggers which facilitate circuit's adaptation for goals G3 and G2 respectively. (B) Same as in (A), but at three time points along evolution: beginning, middle and end of evolution. (C) as in (A), but for the RNA model. The x-axis is labeled with the parenthesis notation for RNA secondary structure. (D) Genetic triggers of (C) placed on the structure of the evolved MVG RNA molecule.

To detect genetic triggers one must search for genomic positions that vary in a way that is highly correlated to the change in the goals. This means that triggers carry high information content about the current goal. The genetic triggers can thus be detected by evaluating the mutual information between the environment (goal) and the genomic content at each position (see Methods). Since mutual information measures how much the knowledge of one variable reduces the uncertainty regarding the other, the trigger positions are characterized by high mutual information with the environment (Figure 4A). Trigger positions were readily detected for all MVG cases tested. In the RNA model, we find that mutual information is spread amongst more genomic positions than in the logic circuit model. Triggers can still be clearly detected at sites with much higher mutual information than the background. We find that these trigger nucleotides are positioned within the module that they affect, usually in the stem of a hairpin (Figure 4C and 4D). In this respect, the hairpins evolved in MVG differ from hairpins evolved in FG in that a single change in the trigger can cause a flip between an open loop and a closed hairpin.

Over time, under MVG conditions, it is evident that the mutual information between genomes and goals (i.e. environments) gradually becomes focused to a few trigger positions, allowing rapid adaptation when environment changes (Figure 4B). Since trigger positions are small variations that lead to a sizable switch between pre-designed states, they may be considered as a simple example of weak regulatory linkage.

### Evolution of Novelty within the MVG ‘Modularity Language’

So far, we analyzed the adaptation to previously seen goals introduced along MVG evolution history, which highlighted the ability of MVG organism to remember its past. We now turn to novel, previously *unseen* goals, where we test the ability to generalize based on the past.

The main problem is to define what kind of novel goals might be encountered in future environments that are in the same context as the previous environments. Indeed, adaptation of MVG-organisms toward a randomly picked goal results in evolution that is as slow, or even slower, than FG-organisms (Text S1 section 6.5). But a randomly picked goal has no correlation with the past. To address this, MVG evolution offers the possibility of presenting a previously unseen goal which is in the same ‘language’ as previous history.

**Figure 5:**
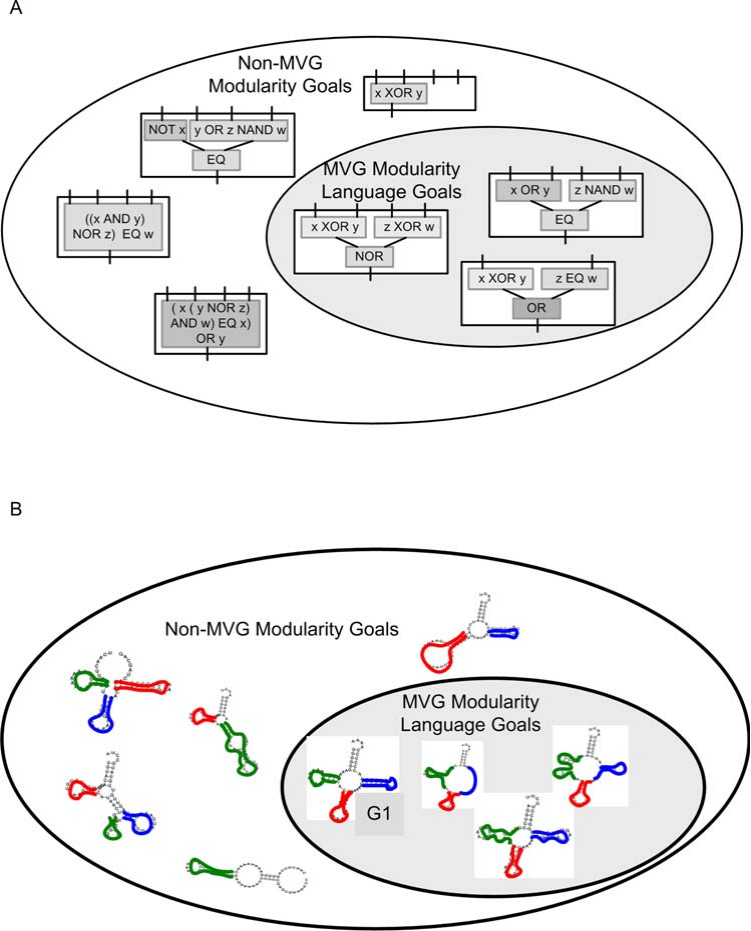
Schematic representation of MVG ‘modularity language’. (A) Logic circuit model, goals within MVG language are of the form *u*(x,y,w,z) = *f*(*g*(x,y), *h*(w,z)). (B) RNA model, goals within MVG language are structures with independent structural modules that correspond in their genomic positions to MVG module.

This language, in the present case of logic circuits, is defined as the set of all goals that can be decomposed in the following way *u*(x,y,w,z) = *f*(*g*(x,y),*h*(w,z)), Figure 5A. In other words, the goals in the language are made of a hierarchy of three functions *f*,*g* and *h*, such that *g* responds to x and y, and *h* responds to the other two inputs w and z, and *f* responds to *g* and *h*. In the case of the RNA model, the language can be defined as the set of all secondary structures with independent structural modules (e.g., hairpin loops, open loops etc.) that correspond in their genomic positions to the modules of the MVG goals (see Methods and Figure 5B).

**Figure 6:**
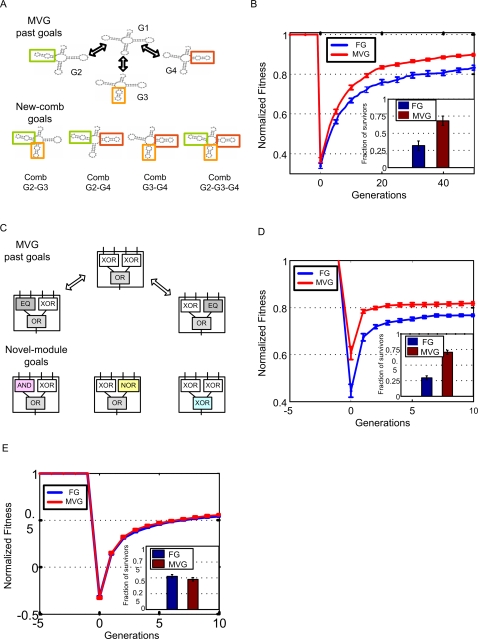
Adaptation towards novel modular goals is more rapid in MVG organisms. (A) An example of *new-comb* goal in the RNA model, where the new goal structure is composed of previously seen sub-structures but in new combinations. (B) Maximal normalized fitness in RNA populations (mean±SE) as a function of generations for *new-comb* structures. The x-axis is generations, where zero is the point where the goal changes to a *new-comb* structure. Initial populations are FG-populations evolved toward G1 and MVG populations taken from the end of the last G1-epoch. Data are from 15 simulations for the four *new-comb* goals of (A). Inset: competition of FG and MVG populations under a *new-comb* goal (following the method of [44]). Initial populations were composed of equal fractions of FG-populations and MVG populations. Data are from 30 competition runs for each of the four *new-comb* goals. (C) *Novel-module* goal in the logic circuit model. In the *novel-module* goal, one of the 2 XORs or the OR operation was changed into a different 2-inputs Boolean function such as AND, NOR or XOR, not seen in the history of the evolution. (D) Maximal normalized fitness (mean±SE) as a function of generations for *novel-module* goals in the logic circuit model. At time zero the goal changes to a *novel-module* goal. Initial populations are as in (B). Data are from 30 simulations in each scenario, for 20 different *novel-module* goals (listed in Text S1 section 6.4.2). Inset: Competition of FG and MVG organisms in a *novel-module* environment. Starting populations were composed of equal fractions of FG and MVG populations. Data are from 30 simulations for 20 *novel-module* goals. (E) Same as in (D) but for non-MVG language goals. Here, the goal is a randomly chosen Boolean function, generated by randomly generating a 4-input 1-output truth table. Goals with a difficulty level similar to that of (D) were chosen, as evaluated (Text S1 section 6.2). Data are for 35 non-MVG language goals. Inset: Competition results as in the inset of (D).

Within this language, we defined two classes of possible future goals which are novel: (a) *New-comb* is a goal that presents previously seen subgoals but in a new combination (Figure 6A) (b) *Novel-module* refers to goals where one of the subgoals is a previously unseen one, while the other subgoals are kept unchanged (Figure 6C). This represents a novelty that is restricted to one of the modules of the goal.

We tested evolution under these two classes of novelty. We find that for both logic circuit and RNA models, MVG populations adapted faster than FG populations when introduced to *new-comb* goals (Figure 6B). We also performed competition experiments in which initial populations were composed of 50% FG-evolved and 50% MVG-evolved genomes. When *new-comb* goals were presented, the descendants of MVG-evolved genomes took over the population in about 68% of the RNA model runs (Figure 6B inset). Logic circuits showed similar behavior, where MVG-genomes took over the population in about 75% of the runs (Text S1 section 6.3).

We also tested *novel-module* goals. Here, the RNA model did not show a significant difference between FG and MVG genomes. However, in the logic circuit model, MVG-populations adapted significantly faster also to *novel-module* goals (Figure 6D). We tested 20 different *novel-module* goals. For example, a novel goal is generated by replacing a XOR module by a previously unseen 2-input Boolean function, such as AND or NOR defined by its truth table (Figure 6C). We find that MVG's outperformance occurred only toward goals within the modularity language. MVG adaptation toward non-modular goals was not significantly different from FG's (Figure 6E).

In competition experiments [44] between FG and MVG genomes toward novel-module goals, populations were taken over by MVG-genomes in about 70% of the runs (Figure 6D inset). In experiments toward randomly chosen goals, populations had equal chance to be taken over by either FG or MVG genomes (Figure 6E inset). We further find that the harder the *novel-module* goal (the more generations needed to solve it ‘from scratch’), the more MVG organisms out-perform FG organisms (see Text S1 section 6.4). These results imply that temporally patterned environments not only lead to a memory of the past goals, but also to generalization: the population learned a language of its history of environments (conditions) that share the same common rules.

### Mechanisms for Enhanced Evolution of Novelty

To examine the mechanisms for enhanced evolution of novelty within the MVG language, we tested three suggested mechanisms proposed in the theory of FV [5] (a) mutations have large effect on their own module. This reduces the number of steps to novelty; (b) mutations have small effect on other modules, a property also called reduced pleiotropy [45],[46]; and (c) mutations have reduced lethality, increasing viable genetic variance in the population and allowing access to higher diversity of potential phenotypes. We quantified the effects of mutations according to these suggestions. The results demonstrate that MVG organisms in the present study follow the first two mechanisms, but not the third.

We begin with the first two mechanisms, and treat the third in the next section. To quantify the effect of mutations on their own module and on other modules, we mutated each of the genome positions that correspond to a given module in the phenotype, and tested its phenotypic effect on its own module and on the other modules. The effect of the mutation was quantified as phenotypic distance: Hamming distance between the structures of subsequences in the case of RNA, and between the series of outputs of the gates (over all input combinations) within each module in the case of logic circuits (see Methods).

The results are summarized in Table 1. Significantly enhanced intra-module change and reduced pleiotropy were found in most cases. The two models differed in the extent of these mechanisms: logic circuits showed more reduced pleiotropy, and RNA structures primarily showed more enhanced intra-module change.

**Table 1:** Intra- and inter-modular effects of mutations.

	Logic circuit model				RNA model			
	Median±SE		p-value	Observed range	Median±SE		p-value	Observed Range
	FG	MVG			FG	MVG		
Intra Module Effect	0.12±0.002	0.14±0.001	<10^−4^	0–0.2	0.28±0.007	0.36±0.005	<10^−9^	0.18–0.64
Inter Module Effect (Pleiotropy)	0.04±0.005	0.01±0.001	<10^−4^	0–0.1	0.057±0.005	0.053±0.005	NS	0.01–0.19

The first row corresponds to the normalized phenotypic effect of a genetic mutation on its own module; the second row corresponds to the normalized phenotypic effect of a genetic mutation within a module on the other modules (pleiotropy). The median±SE are presented for FG and MVG, p-value is as obtained from Wilcoxon rank sum test for equal medians. The range of effects in solution space was obtained by measuring the effects over a large random sample of genomes that solve G1 (see Text S1 section 3).

### MVG Evolution Reduces the Genetic Variance of the Population

**Figure 7:**
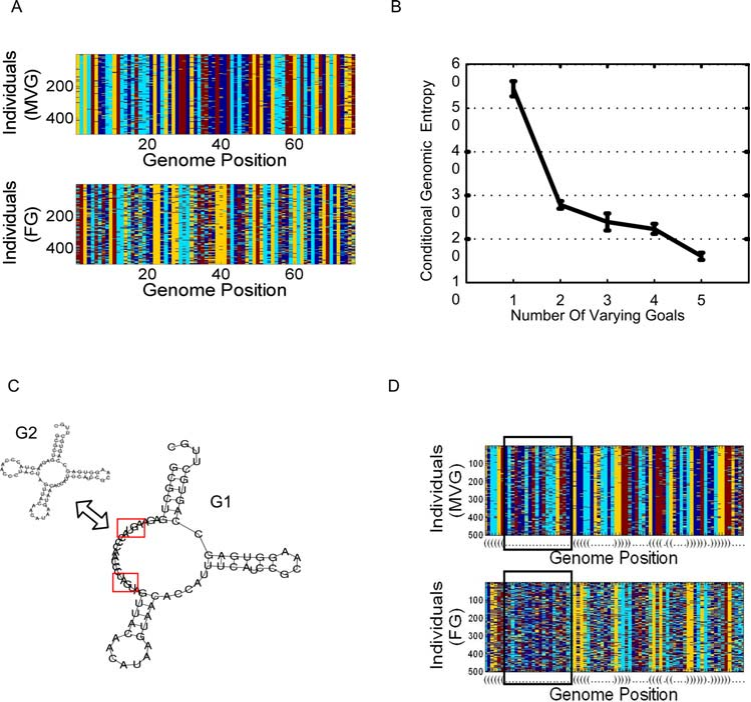
Reduction in genetic variance in MVG evolution. (A) Genomes of evolved RNA populations under FG and MVG scenarios. Each row corresponds to a 76-nucleotide genome of an individual in the population. Color stands for the genomic position content (A,U,G,C). For MVG, the end of the last G1-epoch population is presented. (B) Conditional genomic entropy (mean±SE) as a function of number of goals presented along MVG evolution (x-axis). (C) FG scenario was toward G1. In MVG the goal switched repeatedly between G1 and G2. G2 is a modular variation of G1 with hairpin instead of an open loop in a module corresponding to genomic positions 7 to 26. (D) Genomes of evolved RNA populations with FG and MVG scenarios as described in (C). Note the low variance in MVG populations within the marked region, which corresponds to the genomic positions of the loop region that varies between goals.

We now turn to the third mechanism for novel adaptation proposed by FV theory, associated with an increase in the genetic variance of the populations. We evaluated the genetic variance in a population given its current goal by measuring the conditional genomic entropy (Methods, Text S1 section 11, [47]). In contrast to the suggested mechanism of FV theory, we find that MVG populations display lower genetic variance than FG populations (Figure 7A). The reduction in genetic variance indicates that the rapid adaptation of MVG populations in this study is not due to population diversity but rather in useful potential variation within each individual.

Why do MVG-populations show a lower genetic variance? One possibility is that they evolve to store information about past environments in their genome, placing constraints on the sequence (strong stabilizing selection). To test this, we studied the effect of increasing the number of goals introduced over time in MVG. We find that the more goals (or more precisely the higher the information content in the environment), the lower the genetic variance in the population (Figure 7B). Organisms evolved in constant environment seem to store less information and have higher genomic entropy (Figure 7A).

An additional way to understand the low variance in MVG genomes compared to FG genomes is to consider that the latter are more robust to genomic mutations (see Text S1 section 4.1). Hence, they display more positions in the genome that can be varied without affecting the phenotype. Robustness to mutations thus allows higher genetic variance in the population [48], and conversely, strong constraints on the genome lead to lower genetic variance and sensitivity to mutations in MVG organisms.

As an example for storage of information in the genome and its effect on genetic variance, consider the example of Figure 7C and 7D. Populations of RNA molecules that evolve toward a fixed secondary structure G1 that contains an open loop are found to show high variance in the genomic positions that form the open loop. This is because forming a loop is relativity easy as there are few constraints for base-pairing. On the contrary, populations evolved under MVG environments in which the goal repeatedly switched between G1 and G2 (Figure 7C), show lower variance in the corresponding “loop region”. The evolved MVG loop carries information about its past, and is ready to become a stem by a single ‘trigger’ mutation. The information acquired by the loop is reflected in the pronounced decrease in the variance of that genomic region in MVG populations (Figure 7D).

We note that increase in variance might be expected in more complex models, especially when spatial heterogeneity can allow several metapopulations to exist by using recombination as an efficient adaptation mechanism. High variance may also occur if the genomes can not store the required information (see Text S1 section 10.2 for an example).

### The Phenotypic Neighborhood of MVG Genotype Is Enriched with Novel ‘Useful’ Phenotypes

**Figure 8:**
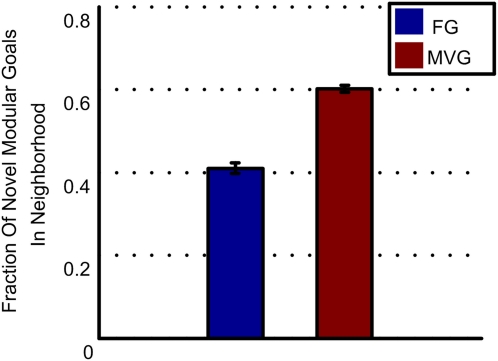
Enrichment of phenotypic neighborhood of logic circuits with novel ‘useful’ phenotypes. Number of novel modular functions divided by the number of all novel functions found in the phenotypic neighborhood of an evolved circuit is shown. Mean±SE is presented for best individuals in MVG and FG populations. Goals were: G1 = (x XOR y) OR (w XOR z), G2 = (x XOR y) AND (w XOR z). For MVG, data are for generations where the goal was G1. Data are from 40 simulations in each case.

We find an additional property of MVG-evolved genomes which helps to overcome barriers to novelty and further reflects the ability to generalize, in the case of logic circuits. In a preceding section, we showed that the MVG phenotypic neighborhood is enriched with phenotypes that are close to previously seen goals. We now turn to possible future goals. We scanned the phenotypic neighborhoods for goals within the same modularity language as previous goals. We find, in the case of logic circuits, that the phenotypic neighborhood of a MVG-circuit is enriched with modular circuits that compute decomposable (modular) functions that are of the form *u*(x,y,w,z) = *f*(*g*(x,y),*h*(w,z)), (Figure 8). In contrast, the neighborhood of a FG-circuit includes more functions that are not decomposable and thus are not within this “modularity language” (see Text S1 section 7). This property was not found for the RNA model.

### Quantitative Measure of Facilitated Variation Shows That It Is Enhanced during MVG Evolution

Finally, we aimed to define a quantitative measure for facilitated variation. A desired measure should capture the two main components of biased variation: (a) the quantity component [41], namely enriching of the phenotypic neighborhood with potentially useful phenotypes which are novel. (b) The quality component: accessing as many as possible different potentially useful novel phenotypes, which are as far as possible in phenotypic distance from the wild-type [49].

We chose a simple FV measure, among other possible choices, which is the product of these two components (see Text S1 section 8.1). The ‘quantity’ component is the probability of forming a potentially useful phenotype which is novel by a single point mutation; the ‘quality’ component is the average phenotypic distance between the wild-type and the potentially useful phenotypes within its phenotypic neighborhood. This measure is then normalized for its corresponding value with respect to non-useful neighboring phenotypes.
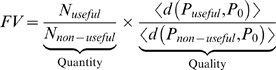
Here, useful phenotypes correspond to phenotypes with the same modular structure as the goals in which the organism has previously evolved. *N_useful_* is the number of neighbors which have a modular phenotype (useful) and are different from the wildtype phenotype (novel), and *<d(P_useful_, P_0_)>* is the mean distance between novel and useful phenotypes and the wildtype. Similar definitions apply for the denominator, where non-useful means phenotypes that do not have the modular structure of previous goals (in the logic circuit model this includes either trivial functions such as an output of all ones or all zeros, or non-decomposable Boolean functions).

**Figure 9:**
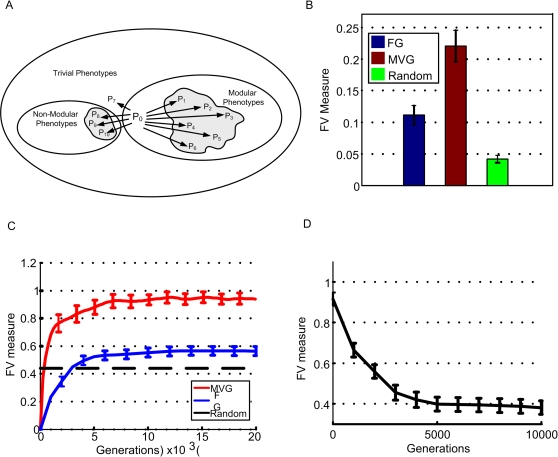
Dynamics of facilitated variation. (A) Schematic presentation of a phenotypic neighborhood with high facilitated variation. Outer ellipse is the phenotypic space, inner ellipses stand for non-trivial Boolean functions that are within (right ellipse), or without the MVG modularity language (left ellipse), P_0_ is the wild-type phenotype and P_1–10_ are neighboring phenotypes. The thickness of the arrow represents the probability of the wild-type to generate P_i_ with one genetic mutation. Length of an edge represents the distance of the phenotypic ‘jump’. High FV corresponds to many long and thick arrows towards the right ellipse. (B) Facilitated variation measure (mean±SE) in RNA model of MVG, FG and a random class of inverse-fold genomes (genomes generated by an algorithm to yield a desired fold) [36] with G1 structure. Data are from 30 simulations in the case of FG and MVG and 200 random genomes. (C) Facilitated variation measure (mean±SE) as a function of generations in logic circuits evolution. Goals were: G1 = (x XOR y) OR (w XOR z), G2 = (x XOR y) AND (w XOR z). For MVG, data are for generations where the goal was G1. Data are from 40 simulations in each case. The random class (dashed line) includes circuits which achieve the goal but were generated by an optimization algorithm rather than by an evolutionary process (see Text S1 section 3.1). (D) Facilitated variation rapidly decays when goal becomes constant over time. Each simulation started from end-of MVG evolution population that had perfect fitness for the goal G1.At the generation marked zero, the population was placed under a FG evolution with the same goal G1, with a selection pressure for minimizing the number of gates [33] (fitness reduction of 0.2/gate for each gate over 10 gates). Mean FV measure (±SE) vs. generations of 500 best-fitness circuits in each population is shown. Statistics are for 30 independent experiments.

According to the formula, an organism with high FV has a high likelihood of forming potentially useful variation and a relatively low probability of varying towards non-useful phenotypic directions (see Methods and Figure 9A).

We find that the FV measure increases with generations under both FG and MVG evolution (Figure 9B and 9C, Text S1 section 8.2). However, it increases significantly more under MVG. The increase under FG evolution seems to result from the increase in robustness (increased probability of generating wild-type phenotype or close to wild-type phenotypes). Finally, we performed experiments in which the initial population consisted of genomes with high FV that were evolved by MVG. We then placed this population under a fixed goal, corresponding to their last seen goal, but presented constantly over time. We find that FV decreased rapidly within a few tens of generations provided there is even a slight selection pressure for small circuit size (Figure 9D, following [33]). This result demonstrates the role of modularly varying goals in preserving facilitated phenotypic variation in the face of more optimal, low FV circuits.

## Discussion

This study quantitatively examined facilitated variation in model systems and demonstrated that it is enhanced in modularly varying environments as compared to constant environments. When the environment varies in a modular fashion (or, more generally, in a systematic manner), it is possible to define feasible future environments that belong to the same ‘language’ as past environment. Hence, one can define a context specific evolvability: the extent to which organisms can generalize and generate novelty that is useful in the context of feasible future environments.

The present results suggest that adaptation to new goals in MVG relies on the evolvability properties of each individual [50]. The evolved organisms are intrinsically designed for a certain class of changes. Organisms that evolve under MVG develop weak linkage implemented by ‘trigger’ genomic positions that elicit a large phenotypic payoff upon minimal genetic investment. The triggers elicit substantial changes in one module and have low effect on other modules (low pleiotropy). The genomes are such that their genomic neighborhood is enriched with a wide range of potentially useful phenotypes – useful in the context of the previous goals ‘learned’ by the organism. Thus, the evolved genomes carry information about past goals. This information effectively prepares the organism for the future, provided that future goals are related to past goals.

The evolution of facilitated variation is time-scale dependant: if goals switched very rarely, it would be equivalent to a succession of FG's. On the other hand, if goals switched too fast, the required information would not have the sufficient time to be assimilated. We find that in the case of the present models, the rate of environmental switching that gives rise to evolvable organisms spans several orders of magnitude [33],[34].

This study employed two different models to study facilitated variation, logic circuits and RNA structures. Importantly, these two models differ in the type of modularity in their goals. RNA goals contained explicit structural modules (e.g. hairpin loops). Every RNA structure that satisfies such goals is modular by definition. In contrast, the modularity in logic circuits goals is implicit. Circuits that satisfy a modular goal can have either a modular circuit structure or a non-modular one. Modular circuit structures are in fact much more rare, and tend to evolve only under MVG, where switching between goals with shared modules constrain the circuits to evolve structural modules [33]. This difference between RNA and logic circuit models may underlie the fact that logic circuits showed a very strong enhancement of facilitated variation in MVG compared to fixed goals, whereas RNA model had a more modest enhancement. These two models are approximations to different aspects of biological design: Cell signaling and regulation networks that compute responses to signals are more analogous to the circuit model, whereas molecular structures are akin to the RNA model.

What happens if goals vary over time but in a *non-modular* fashion? We find that an environment that varies between randomly chosen goals typically causes confusion, where no good solution is found that can rapidly adapt to both goals. It is possible, however, to find pairs of goals which are not modular and yet which have solutions that are only a few mutations away from each other. In other words, goals whose neutral networks happen to come very close at a certain point. Here, genomes evolve that show rapid adaptation each time that the goal switches, but do not have modular phenotypes. However, it is hard to define facilitated variation towards novel goals in this case, since one can not define the future goals that are in the same ‘language’. Adaptation to novel goals is generally very poor (see Text S1 sections 2.1 and 6.5). In summary, evolution under non-modular varying environments might lead in certain cases to memory but not to generalization.

Modularly varying goals seem to enhance facilitated variation because of two main effects (i) they greatly improve the chances for the existence of solutions for the different goals that are close in genetic space (because the same modules need only be rewired by a few mutations) (ii) they offer the possibility of learning not only past goals, but also generalize to future goals as long as they are made of the same subgoals or with the same division into modules as previous goals. Finally, we note that facilitated variation comes with a cost: organisms are less optimal to the current goal than they might have been. For example, logic circuits that have high FV are usually composed of more logic gates than the optimal circuits that evolve if this goal is kept constant for very long times. Modularity, genetic triggers, and storage of information about the past in the genome, seem to demand more genes than is absolutely required to solve the problem. Extreme optimality to present environments is sacrificed to provide readiness to future ones.

Organisms or molecules that are under constant conditions [51],[52] are predicted by the present theory to lose their FV design, and become less evolvable. One may test this prediction by comparing organisms that evolved in varying and relatively constant environments [51],[52]. A further prediction is that any fluctuation in the system (such as molecular noise [53] or thermal fluctuation) would result in an output that is channeled in potentially useful directions.

In summary, the present study aimed at studying facilitated variation in simple model systems. Populations evolved under systematically varying conditions were found to exhibit not only a memory of past goals but were also able to generalize to new conditions that are in the same language as previous conditions. Adaptation to useful novel goals was enhanced by organisms that have learned the shared subgoals that existed in past environments and are therefore likely to be encountered in future environments. Several elements of facilitated variation theory, such as genetic triggers, modularity, and reduced pleiotropy of mutations seem to evolve spontaneously under these conditions. It would be interesting to study the evolution of additional FV mechanisms such as exploratory behavior and body-plan compartmentalization using more elaborate models with hierarchical designs and developmental programs.

## Methods

### Genetic Algorithm

We used a standard genetic algorithm [54],[55] to evolve combinatorial logic circuits and a structural model of RNA. The settings of the algorithm were as follows: a population of *N*_pop_ individuals was initialized to random binary genomes of length *B* bits (random nucleotide sequences of length *B* bases in the case of RNA, in the main examples *B* = 76 for the RNA and *B* = 104 for logic circuits). In each generation, *N*_pop_ individuals were selected with repeats from the previous generation according to a probability that exponentially scales with their fitness (selection strategy, see Text S1 section 1). Pairs of genomes from the selected individuals were recombined, using crossover probability *P*_c_ (*P*_c_ = *0.5* for the circuits model; *P*_c_ = 0 for the RNA model) and then each genome was randomly mutated (mutation probability *P*_m_ = 0.7/*B* per locus per genome). The present conclusions for the logic circuit model are generally valid also in the absence of recombination (*P*_c_ = 0). The present results were based on simulations of a population of size *N*_pop_ = 5000 evolved for *L* = 10^5^ generations for the circuit model, and a population of *N*_pop_ = 500 evolved for *L* = 10^5^ generations for the RNA model. These population sizes were empirically found to serve as minimal values for many of the presented effects, which seem to apply also for larger population sizes. For statistical analyses we considered only simulations that ended with maximal fitness of 1 within the predefined generation limit *L*. Similar conclusions were found when analyzing all runs.

### Logic Circuits Evolution (Model 1)

Circuits were composed of up to twelve 2-input NAND gates. The binary genome coded for the circuit wiring as described in [33],[34],[54],[55]. Self loops and feedback loops were allowed. Goals were 4-input 1-output Boolean functions composed of XOR, EQ, AND, and OR operations. The goals were of the form *u*(x,y,w,z) = *f*(*g*(x,y),*h*(w,z)), where *g* and *h* were 2-input XOR or EQ functions, and *f* was an AND or an OR function [34]. Each Boolean function can be represented as a truth table, where each row represents a different combination of inputs values (0 or 1), and the relevant output value (again 0 or 1). Thus each goal can be uniquely defined by the output column vector. The fitness of each circuit was defined as the fraction of correct outputs over all possible inputs. In the MVG simulations the goals were modularly related by changing the functions *f*,*g* or *h*. The goal changed over time in a probabilistic manner every *E* = 20 generations.

### RNA Secondary Structure (Model 2)

We followed the work of Schuster [42] and Ancel and Fontana [12] and used standard tools for structure prediction available at http://www.tbi.univie.ac.at/RNA/, and the “tree edit” structural distance [56]. The goals were secondary structures of length 60–90 nucleotides such as the *Saccharomyces cerevisiae* phenylalanine tRNA and synthetic secondary structures composed of three hairpins (for the full list of structures see Text S1 section 1.2). In MVG, the modular changes were applied by modifications of single hairpin at a time (such as changing the shape of the hairpin to an open loop). Goals changed every *E* = 20 generations (unless otherwise noted).

### Normalized Fitness

Normalized fitness in Figures 3A, 6B, 6D, and 6E is defined as 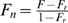, where *F* is the maximal fitness in the population and *F_r_* is the average maximal fitness of a population of *N*_pop_ random genomes. Normalized fitness *F_n_* = 1 means a perfect solution to the goal, and *F_n_* = 0 means a solution that is as good as expected in a random population of the same size. For the purposes of computing the best fitness *X* of a genetic neighborhood of a given system with phenotype P, as in Figure 3B and 3C, we used a normalization in which *F_r_* is the value of *X* averaged over *N*_pop_ samples taken from genomes with the same phenotype P. In the case of logic circuits, genomes with the phenotype P were obtained by simulated annealing optimization algorithm which produced genomes that satisfy the desired goal. In the case of RNA structures, genomes with phenotype P were generated using a standard inverse fold algorithm [36]. The normalized fitness of the genetic neighborhood is 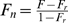.

### Quantitative Measure of Genetic Variance

Following Adami et al. [48], genetic variance was measured using entropy H computed as follows. In a RNA genome of length *B*, each position can hold one of the 4 possible nucleotides with the probabilities: P_i,j_ where i = 1..*B* and j = {C,G,A,U}. The entropy of position i is H_i_ = −ΣP_i,j_log(P_i,j_). The maximal entropy per position (using logarithm of base 4) is 1, which occurs when the nucleotides distribution at that site is uniform. Perfectly conserved positions have zero entropy meaning that they contain maximal information (see Text S1 section 11.1). The nucleotide probabilities for each genomic position were computed from the population genomes. The genetic entropy is the sum of the entropies of all positions. We note that this is only an approximation of the full genomic entropy since we ignore the epistatic relations between positions. It is also important to note that this measure is not the marginal genomic entropy but the conditional entropy of the genome given its current environment (for FG, the two measures coincide). For an example, see Text S1 section 10.1.

### Detection of Genetic Triggers

In order to detect the genetic triggers in a genome, we computed the mutual information I between target goal T and specific genomic site i, X_i_, as I(X_i_,T) = H(X_i_)−H(X_i_ |T) where H is the entropy per site as described above [see Text S1 sections 10 and 11]. Triggers are defined by the positions with the highest mutual information (I) between goal and genomic contents.

### Intra- and Inter-Modular Effects of Mutations

To define the effects of mutations on phenotype modules, we first computed the modules in each phenotype. For RNA this was based on the modular partition of the structure (into hairpin loops etc.), and in logic-circuits, modules were defined using the Newman-Girvan algorithm [57]. We then measured the effects of each possible genomic mutation on the phenotype of its own module, and on the phenotype of all other modules. In the RNA model, the effect of mutations on the phenotype of each module was evaluated by the distance *d* between the wild-type and the mutant structure in each module (Hamming distance between the string representations of the secondary structure [58]). In the case of logic circuits, the output series of each gate was evaluated, and the Hamming distance *d* between the mutant and wild-type was evaluated for each gate. *Intra-module* effects of mutations were the mean of all changes in the same module as the mutated gate, and *inter-module* effects of a mutation was the mean effect on the output of all gates in all other modules. The physical ranges of those effects were estimated by analyzing samples from the solution space (obtained by optimization algorithms).

### Logic Circuits Modularity

To quantify the modularity of a network we used the normalized *Q_m_* measure of Kashtan et. al. [33],[51].

### Definition of Phenotypic Distance

#### Logic circuit model

A logic circuit computes Boolean function of inputs, thus the phenotype can be described as a truth table (in our model, the goal function was 4-input, 1-output). We define the phenotypic distance of two circuits, as the Hamming distance between the corresponding output columns of two truth tables, i.e. fraction of different entries produced by the two circuits. In cases in which the output of the gate/circuit was time-dependent (oscillatory), we simulated the output of the gate/circuit over a window of 20 time-points. The final phenotypic distance was obtained by averaging the truth tables-distances over all time-points, and taking the best result out of all possible frames with a sliding window of 1-time point.

#### RNA secondary structure model

The phenotype of RNA sequence is a secondary structure that can be represented as a string of left and right parenthesis [36]. We used the ‘tree-edit’ distance [56] to compute the phenotypic distance between two legal structures (i.e. structures with balanced left and right parenthesis, where the number of left parenthesis is always larger or equal to the number of right parenthesis when reading the string from left to right). When measuring the phenotypic change in a certain module, ‘tree-edit’ distance can not be applied (since it operates on two legal structures, and sub-structure in a mutant genome is not necessarily legal). In such cases, we measured the Hamming distance between the two parenthesis sub-strings.

### Definition of Potentially Useful Phenotypes

#### Logic circuit model

A potentially useful phenotype in the present context is a decomposable (i.e. modular) Boolean function of the form: *u*(x,y,w,z) = *f*(*g*(x,y), *h*(w,z))where *f*,*g* and *h* correspond to any 2 input, 1-output Boolean function, such as: AND, NAND, OR, XOR, EQ. Trivial cases such as *u*(x,y,w,z) = 0 or *u*(x,y,w,z) = x were not considered.

#### RNA secondary structure model

A potentially useful neighboring structure in the present context is a structure with independent structural modules that correspond in their genomic positions to the wild-type modules. To define this, consider a phenotype P′ in the phenotypic neighborhood of sequence S_0_, with MFE structure P_0_ (the wild-type structure), we say that P′ is a viable phenotype if: (i) P′ has legal sub-structures (legal parenthesis strings) at the genetic positions correspond to P_0_'s modules and (ii) The genomic positions that correspond to distinct inter-module locations (for example, positions between module 1–2 and positions between modules 3–4) in P_0_, do not base-paired with each other in P′.

## Supporting Information

Text S1 Supporting Information. Includes additional detailed examples and analysis.
(3.02 MB DOC)
