# Supplementary material for: Facilitated Variation: How Evolution Learns from Past Environments To Generalize to New Environments
Source: PLoS Comput Biol. 2008 Nov 7;4(11):e1000206. doi: 10.1371/journal.pcbi.1000206 (PMC2563028; doi:10.1371/journal.pcbi.1000206)
Supplement: Text S1 — Supporting Information. Includes additional detailed examples and analysis. (3.02 MB DOC) [file pcbi.1000206.s001.doc]

**Facilitated variation: How evolution learns from past environments to generalize to new environments**

Merav Parter*, Nadav Kashtan*, Uri Alon

Dept. Molecular Cell Biology

Weizmann Inst. of Science, Rehovot Israel 76100

**Supporting Information**

This supporting information is organized as follows:

**1. Description of two model systems used in this study**

1.1 Combinatorial logic circuits (model-1)

1.2 RNA secondary structure (model-2)

**2. Different scenarios of varying environments**

2.1 Non-modularly varying environments in logic circuit model

2.2 Analysis of thermally fluctuating environments in RNA model

**3. Sampling the solution space**

3.1 Simulated annealing (model-1)

3.2 Inverse-fold algorithm (model-2)

**4. Dynamical properties of RNA evolution with FG and MVG scenarios**

4.1 Genetic and thermodynamic robustness

4.2 Melting behavior and G-C content

**5. Detailed analysis of MVG adaptation toward previously-seen goals (model-2)**

5.1 Mutational analysis

5.2 Similarity between genetic and thermodynamic neighborhoods ("Plastogenetic congruence")

**6. Adaptation toward novel goals**

6.1 Classes of novel goals

6.2 Controlling complexity of novel goals

6.3 Methods for comparing populations’ evolvability

6.4 Adaptation toward *novel-module* goals

6.4.1 The relation between speedup and goal complexity

6.4.2 List of novel-module goals studied

6.5 Adaptation toward *random novel* goals

**7. Complete characterization of the phenotypic neighborhood (model-1)**

**8. Facilitated variation analysis**

8.1 Facilitated variation measure

8.2 Evolution of facilitated variation on a neutral network

**9. Quantitative measure of mutational modularity (model-1)**

**10. Conditional entropy measure**

10.1 How to measure conditional entropy from population genomes?

10.2 Genetic variance for large number of varying goals

**11. Characterization of evolved genome positions**

11.1 Conserved positions

11.2 Random-drift positions

11.3 Genetic triggers

**1. Description of two model systems used in this study**

We used standard genetic algorithms (GA) to evolve two well-studied models. The settings of the experiments were as follows: A population of *N*_pop_ individuals was initialized to random Boolean genomes. In each generation all the individuals in the population were evaluated for their fitness *F*. Next generation individuals were selected with a probability that increases with their fitness (with replacements): individual i was selected with probability $e^{t*Fi}/\sum_{i}^{Npop} e^{t*Fi}$ (*t* = 30 in our simulations, *t* = 20 gives similar results) [1]. Pairs of random individuals were then recombined (using crossover operators) with probability *P*_c_, and randomly mutated with probability *P*_m_ = *P*_T_*/B* per genomic position per genome per generation (*B* is the genome length). Simulations were run for *L* generations using a 64-CPU SUNGRID computer cluster. Data shown includes only runs that reached at least one perfect solution to the goal. Similar conclusions were found when analyzing all runs.

**1.1 Combinatorial logic circuits (model-1)**

Detailed description of genome organization and fitness calculations is provided in [2,3]

Genetic algorithm settings^*^: *B* = 104; *N*_pop_ = 5000; *P*_c_ = 0.5 or 0; *P*_T_ = 0.5; *L* = 1x10^5^; *TH* = 1.

*Data shown for *P*_c_ = 0.5, *P*_T_ = 0.5, results hold for *P*_c_ = 0, *P*_m_ = 1, but are slightly more significant with the former setting.

Modularly Varying Goal scenarios:

To generate modularly varying goals, we considered goals of the form *u*(x,y,z,w)=*g*(*f*(x,y),*h*(z,w)) , which can be thought of as a hierarchy of three subgoals each with two inputs: the first computes a function of x and y, the second computes a function of z and w, and the third combines these two. For example, a goal of this form is G1 = (x XOR y) OR (w XOR z).

To perform MVG, we considered two kinds of modular variations of G1:

1. Changing either the of the functions *f* or *h*, for example, changing one XOR module into an EQ

G2 = (x **EQ** y) OR (w XOR z)

G3 = (x XOR y) OR (w **EQ** z)

2. Changing the function *g* (the sub-goal ‘combination’ function)

G4 = (x XOR y) **AND** (w XOR z)

The goals switched as a random walk on the graph described in Fig. 2A. Goal switched every *E*=20 generations. We also considered other MVG scenarios where the OR function of G1, G2 and G3 is replaced by an AND.

Facilitated variation analysis: The analysis was based on 30 simulations for each of the tested scenarios. In MVG, analysis provided in main text was preformed on end-of-G1-epoch populations. The entire analysis holds also in the cases of all G≠G1 goals.

**Figure S1**

**
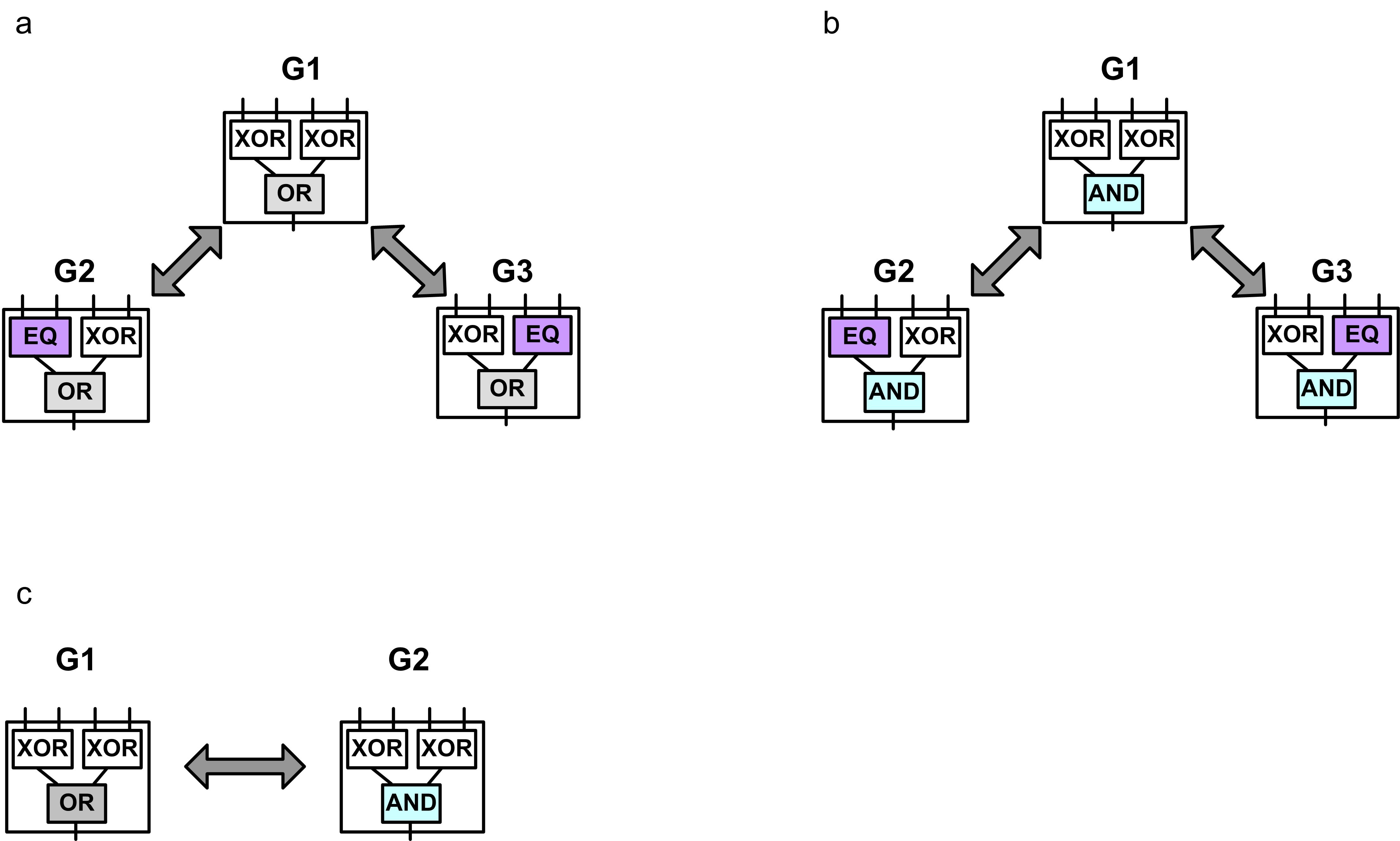
**

**Figure S1.** Schematic presentation of scenarios analyzed. Analysis was performed for each goal of scenarios a-c.

**1.2 RNA secondary structure (model-2)**

Detailed description of the RNA model is provided in [3].

Modularly Varying Goal scenarios:

To generate modularly varying goals, we considered hairpins and general enclosed stems sub-structures as structural modules**.** Our main goal (G1) was a natural tRNA secondary structure (phenylalanine tRNA of *S. cerevisiae*). Modularly varying variants of G1 were obtained by changing each of the three hairpins to open loops (Fig. 2B). The goal switched every *E* = 20 generations. Note that each switch imposed a change of a single ‘module’ in the goal.

For this example (Fig. 2A) the settings of GA were as follow:

*B* = 76; *N*_pop_ = 500; *P*_c_ = 0; *P*_T_  = 0.7; *L* = 2$\times$10^4^.

We also considered other MVG scenarios, as described in Figure S2.


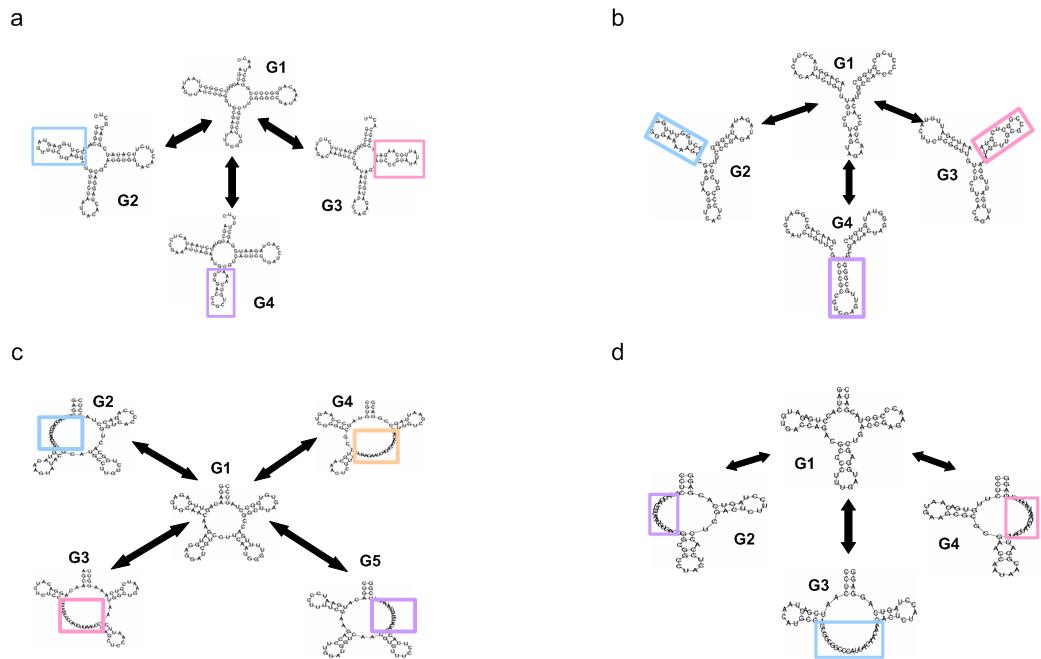
**Figure S2**

**Figure S2**. Scenarios of modularly varying goals in the RNA model, a schematic view. The goal switched during evolution in a probabilistic manner as a random walk on the graph, going from G1 to one of its alternatives, and then back to G1, etc.

Genetic algorithm settings: *B* = 80;61;74; 64 nucleotides; *N*pop = 500; *P*c = 0; *P*T = 0.7; *L* = 10^4^ ;  *E* = 20;30;20;20.

Facilitated variation analysis: The analysis was based on 30 simulations in scenario 1 and 15 simulations for the other scenarios. In MVG, analysis was preformed on end-of last G1-epoch populations.

**2. Different scenarios of varying environments**

**2.1 Non-modularly varying environments in logic circuit model**

In addition to FG and MVG scenarios we also examined evolution with non-modularly varying goals. During this evolution, the environment was changed in a non-modular fashion by switching between non-modularly related goals such as G1 and G2, listed below. Here, we mean non-modular in the sense of not separable to three functions *f*,*g* and *h* as defined in section S1.1. For example, G1 is modular but G2 is not

G1 = (x XOR y) AND (w XOR z)

G2 = (x AND (w NAND z)) OR (w NOR z)

Most choices of G2 lead to ‘evolutionary confusion’ of evolution when temporal switching occurs, in which no good solution is found that can rapidly adapt to both goals. To avoid this and provide a more stringent comparison, we chose G2 goals that have close solutions to G1. That is G2 goals whose neutral networks come close to the G1 neutral network. To find such a neighboring goal G2 which is a non-modular variant of G1, we scanned the phenotypic neighborhood of genomes sampled from the G1 neutral network (see section 3), and ranked the Boolean functions according to their appearance in the set of neighboring phenotypes. G2 was chosen such that it had an approximately median ranking and was not a trivial function or a modularly decomposable one. We denote this scenario, where the goal is periodically switched between two neighboring (non-modularly related) goals, as **N**eigh**b**oring **V**arying **G**oal evolution (in short, NBVG).

We find that the evolutionary dynamics of NBVG is very similar to that of MVG, with respect to the rapid adaptation when environment changes. The design and the mechanisms that underlie this rapid adaptation are equivalent to that of MVG and include the location of genomes at the border of the neutral networks and the evolution of small number of genetic triggers (Fig. S3).

**Figure S3**

**
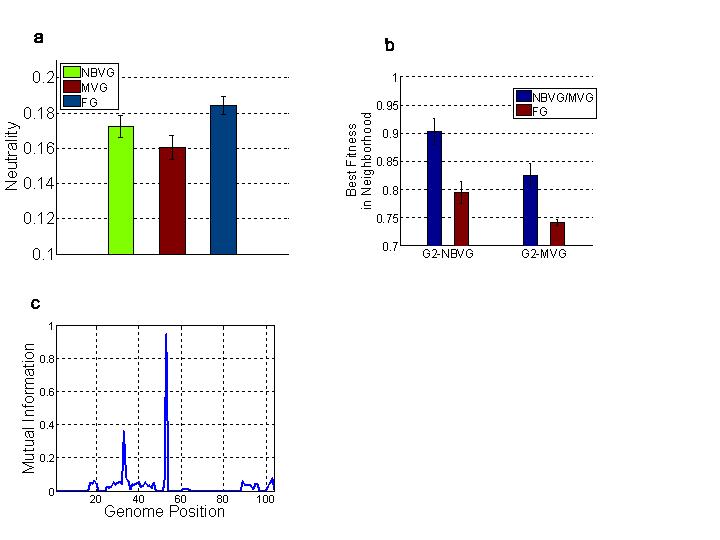
**

**Figure S3.** Common features of MVG and neighboring-varying-goals (NBVG) evolutions. Goal were: G1 = (x XOR y) AND (w XOR z), G2-NBVG = (x AND (w NAND z)) OR (w NOR z) and G2-MVG = (x XOR y) OR (w XOR z). FG was evolved toward G1. MVG goals switched between G1 and MVG-G2 every 20 generations. NBVG evolution was the same as MVG but with G2-NBVG instead of G2-MVG. (a) Maximal fitness (mean ± SE) for G2 in the phenotypic neighborhood of evolved logic circuits. For M/NBVG, genomes from the end of the last G1-epoch population were analyzed. (b) Neutrality (mean ± SE) of evolved circuits is presented for the three scenarios. Neutrality was defined as the fraction of 1-mutant circuits that compute the same Boolean function as the wild-type (G1). (c) Evolution of genetic triggers in NBVG evolution. Mutual information (y-axis) between the environment (goal) and the genomic content in each position (X–axis).

Despite the similarity in dynamics, NBVG and MVG show differences with respect to facilitated variations measures: MVG evolution evolves modular designs which are characterized with a biased variation toward decomposable Boolean functions. In contrast, NBVG evolution imposed no consistent context and thus a language of potentially useful phenotypes could not be defined straightforwardly. The evolved NBVG circuits were even less modular than FG circuits (Fig. S4a), with very low facilitated variation in the context of MVG evolution (Fig. S4b). Our results, therefore, demonstrate the connection between the mode of changing environment and the nature of the evolved variation. Environments that change in a non-random modular fashion enhance evolution of non-random, facilitated-variation. Environments that change in a more random fashion evolve organisms with a more random, un-biased phenotypic variation, with very low FV measures. Finally, environments that do not change at all (FG) evolve organisms with a medium level of facilitated variation (due to increased in robustness).


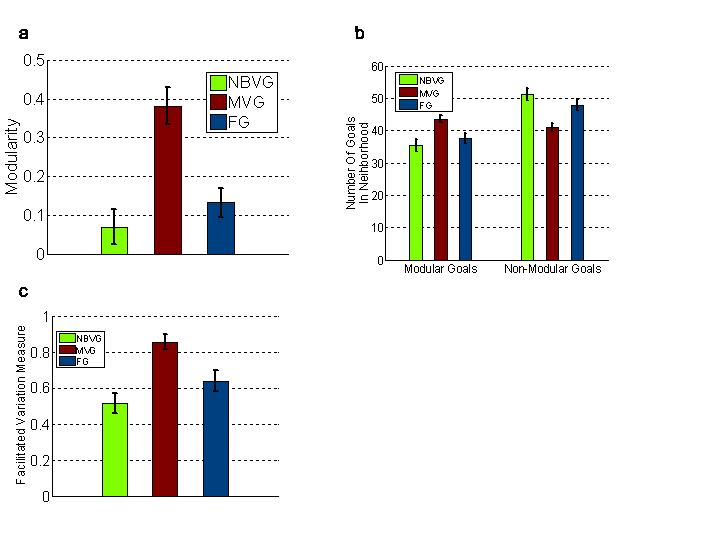
**Figure S4**

**Figure S4.** Distinct features of MVG and NBVG evolutions. (a) Averaged modularity (Qm) of genetic neighboring circuits. Mean ± SE is presented for each scenario. (b) Number of modular and non-modular goals in the phenotypic neighborhood of evolved circuits. (c) Facilitated variation measure (based on MVG context). For NBVG/MVG, genomes are from the end of the last G1-epoch populations. In each scenario, 30 simulations were analyzed.

**2.2 Analysis of thermally fluctuating environments in RNA model**

RNA model offers two possible scenarios of non-modularly varying environments. In the first scenario, the goal is switched between non-modularly related goals (as for logic circuit model). In the second scenario, thermal fluctuations are introduced along evolution (for example, by changing the folding temperature). We analyzed both types of varying environments. Since the first NBVG scenario yielded qualitatively the same results as logic circuit model, we discuss only the second scenario.

Description of simulations with thermally fluctuating environments:

Every *E* = 20 generations, the folding temperature of RNA population was changed according to the uniform distribution in the range 10-90^o^C,

With the settings: *N*_pop_ = 500, *P*_c_ = 0, *P*_T_ = 1, *L* = 1x10^4^.

We find that under thermal fluctuating environment the evolved genomes show thermodynamic robustness - they maintain their secondary structure over a wide range of temperatures. However, the evolved robustness came with a cost: The diversity of the thermodynamic and the genetic neighborhoods was significantly decreased. The resulted genetic canalization led to a point of lock-in, where evolution mostly ended with sub-optimal solutions and failed in finding prefect ones (Fig. S5). Fontana et. al. [4] analyzed these effects in a plastic model of RNA evolution. The thermal fluctuating environment, analyzed herein, is effectively the same as evolution with a direct selection against plasticity. Both cases lead to evolutionary lock-in, and to high thermal and genetic robustness.

**Figure S5**

**
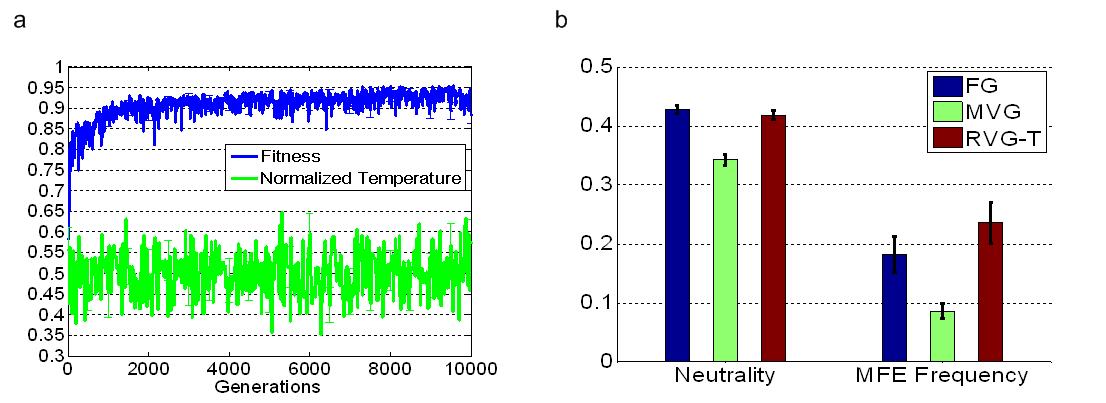
**

**Figure S5**. RNA evolution under thermally fluctuating environments (denote as RVG-T). Shown are maximal fitness in the population (mean ± SE) and the normalized folding temperature (divided by 100) as a function of generations where the target structure is G1 (tRNA). (b) Genetic neutrality (mean ± SE) and a thermodynamic stability measure (MFE frequency) of evolved RNA genomes are presented for the three scenarios. Neutrality was defined as the fraction of 1-mutant genomes with same structure as the wild-type (see table S1). For MVG, data are for epochs where the goal was G1. Data are for 20 simulations in each scenario.

**3. Sampling the solution space**

**Motivation:** In the present study, we mainly focused on comparing the level of facilitated variation of MVG genomes to that of FG genomes. One question that may arise is what is the effect of FG evolution on facilitated variation. In other words, does natural selection by itself (FG scenario) facilitate, to some extent, the variation of the individual?

We address this question by considering a third class of organisms with the same phenotype as FG and MVG evolved organisms. However, those organisms were not evolved by means of evolutionary simulations (i.e. weren’t selected by a process akin to natural selection) but instead were obtained by applying optimization algorithms (as described in section 3.1 and 3.2). In contrast to FG and MVG populations which were restricted to certain regions of the neutral network, the non-evolved organisms can be considered as a sample from the neutral network of the target goal. Indeed we find that these organisms span many different regions of the neutral network.

We compared FG-evolved organisms to this sample. We find that FG has higher level of facilitated variation compared to non-evolved but high fitness organisms. We find that the increase in facilitated variation in FG-organisms is due to increase in genetic robustness (thus increasing the probability of generating a wild-type phenotype or a close to wild-type phenotype upon genetic mutations). The non-evolved organisms class has another important role: it defines the possible realms of values to the different measured properties an organism can have for a given neutral network. We then were able to analyze to what extent FG or MVG evolution affected these values (Fig. S6).

**3.1 Simulated annealing (model-1)**

A simulated annealing (SA) algorithm [5] was applied to obtain 5000 (*N*_pop_ in logic circuit model) different solutions to G1. It’s noteworthy that this sample might not be an unbiased random sample of a neutral network. A bias might be introduced by the fitness landscape of the goal, since solutions surrounded by valleys are less likely to be sampled. For description of SA setting see [3].

SA settings: *B* = 104 bits, *N*_pop_ = 1, *P*_c_ = 0, *P*_T_ = 0.7, *t* = 5, half-time=1000.

**3.2 Inverse-fold algorithm (model-2)**

Inverse-fold algorithm was applied to obtain a set of 500 genotypes (*N*_pop_ in RNA model) with a desired target shape [using RNAinverse, see Ref [6]]. The genotype-phenotype RNA mapping introduced a bias in this case as well, yet, this method is commonly used to obtain a background class to which artificial or natural genomes are compared to [4,7].

**Figure S6**

**
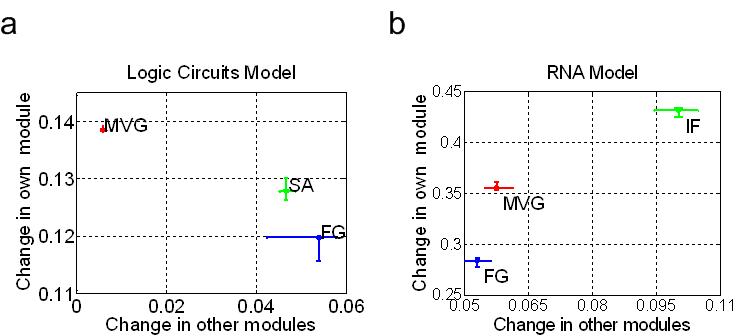
**

**Figure S6**. Intra- and inter-module effects of mutations. The y-axis corresponds to the phenotypic effect of a genetic mutation on its own module; the x-axis corresponds to the phenotypic effects on the other modules (pleiotropy). The median ± SE are presented for FG, MVG and for perfect solutions obtained by optimization algorithms **(a)** Logic circuit model, SA stands for solution found by a simulated annealing algorithm. **(b)** RNA model. IF stands for inverse-fold genomes. Data are from 30 simulations in each scenario. 10 best individuals were analyzed in each MVG/FG population (SE was evaluated using a bootstrapping method).

**4. Dynamical properties of RNA evolution with FG and MVG scenarios**

**4.1 Genetic and thermodynamic robustness**

**Table S1. Definition of genetic and thermodynamic properties in** RNA model

|  | Property | Definition |
| --- | --- | --- |
| **Genetic Properties** | *Neutrality* | Fraction of 1-mutant neighborhood that preserves the wild-type phenotype |
|  | *Neighborhood Diversity* | No. of different structures in 1-mutant neighborhood |
|  | *Neighborhood Diameter* | Average structural distance in the 1-mutant neighborhood |
| **Thermodynamic Properties** | *MFE Frequency* | The probability to be in the Minimal Free Energy configuration. |
|  | *Neighborhood diversity* | No. of configurations near the MFE shape |
|  | *Neighborhood Diameter* | Expected structural distance in the thermodynamic ensemble |

**Figure S7**

**Figure S7.** Summary analysis of genetic and thermodynamic properties of table S1. Mean ± SE of each property is shown. Best 10 genomes from the end of the last G1-epoch population were analyzed. Data are from 30 simulations in each scenario.

**4.2 Melting behavior and G-C content**

**Figure S8**

**
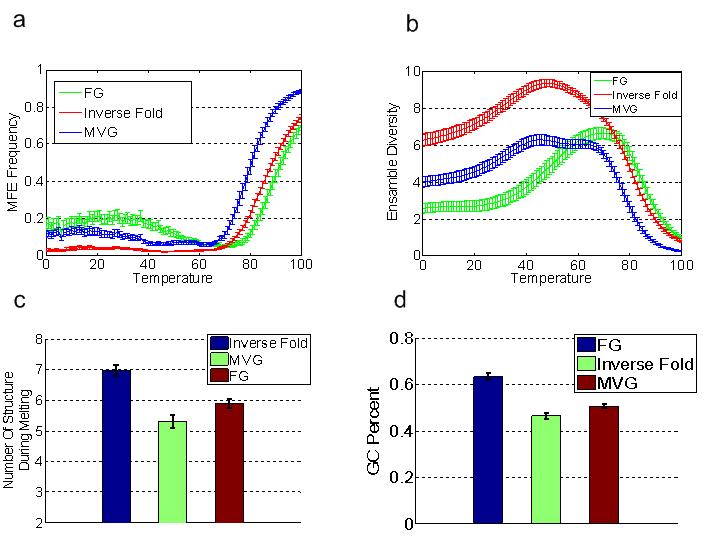
**

**Figure S8.** Melting Behavior and G-C content **(a)** MFE frequency (Mean ± SE) as a function of folding temperature. **(b)** Mean (± SE) of thermodynamic ensemble diversity as a function of folding temperature. **(c)** Number of distinct MFE structures along melting (*T* $\in$0-100^o^C). This property is not general as it was not found for all scenarios analyzed. Data are shown for the main example of tRNA goals. **(d)** MVG evolved structures are less rigid. Fraction of G-C bp in folded structure (Mean ± SE). Best 10 genomes from the end of the last G1-epoch population were analyzed. In each scenario, 30 simulations were analyzed. Inverse fold is a collection of *N*_pop_ genomes with the same structure as MVG and FG evolved genomes (see section 3.2).

**5. Detailed analysis of MVG adaptation toward previously-seen goals (model-2)**

**5.1 Mutational analysis**

We computed the consensus genome of MVG population (end of last G1 epoch), and classified its positions (Fig. S9a) into three classes: a. *beneficial position*, position that might increase the fitness of the genome upon one genetic mutation, b. *neutral position*, position that is not beneficial and that at least one mutation in this locus is neutral (i.e. maintains the MFE shape) and c. *deleterious position*, position that is not beneficial or neutral, i.e. position that will reduce the fitness upon all possible three base substitution.

We find that about 80% of the deleterious mutations with respect to G1 (the current evolutionary goal of the population), are in fact beneficial with respect to the past goals G≠G1, (Fig. S9a). We also find that the beneficial directions toward a given past goal are located within the specific module that past goal effects (Fig. S9b).

**Figure S9**

**
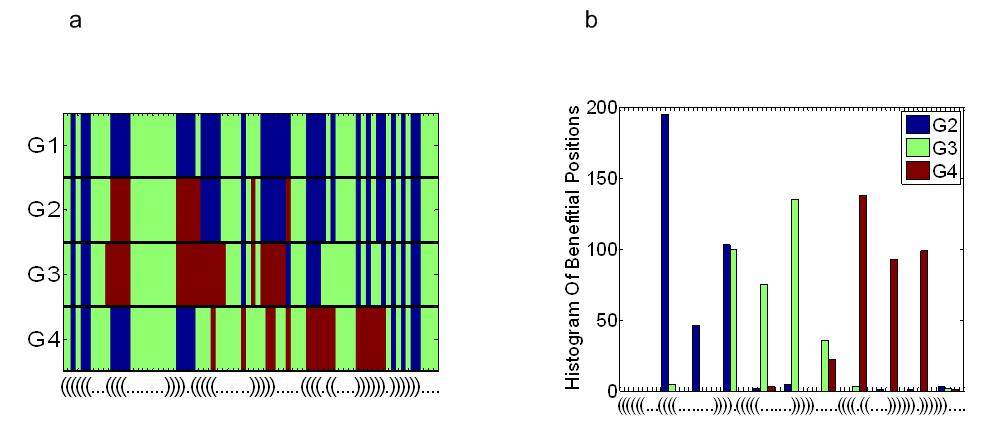
**

**Figure S9.** In MVG, most of the deleterious mutations with respect to G1 are beneficial with respect to past goals **(a)** the positions of a consensus sequence are colored according to their types with respect to G1-G4 goals. Legends: green-neutral positions, blue-deleterious positions, red-beneficial positions. The analyzed population is the end of last G1-epoch population taken from a particular MVG evolution experiment. The x-axis is labeled with the parenthesis notation of G1 structure. **(b)** Histogram of beneficial positions on MVG genomes with respect to past goals. Best 10 genomes from the end of the last G1-epoch population were analyzed. Data are for 30 simulations.

**5.2 Similarity between genetic and thermodynamic neighborhoods ("Plastogenetic congruence")**

RNA Structures that are very close to past goals can be found in the genetic and the thermodynamic neighborhoods of MVG sequence.

**Figure S10**

**
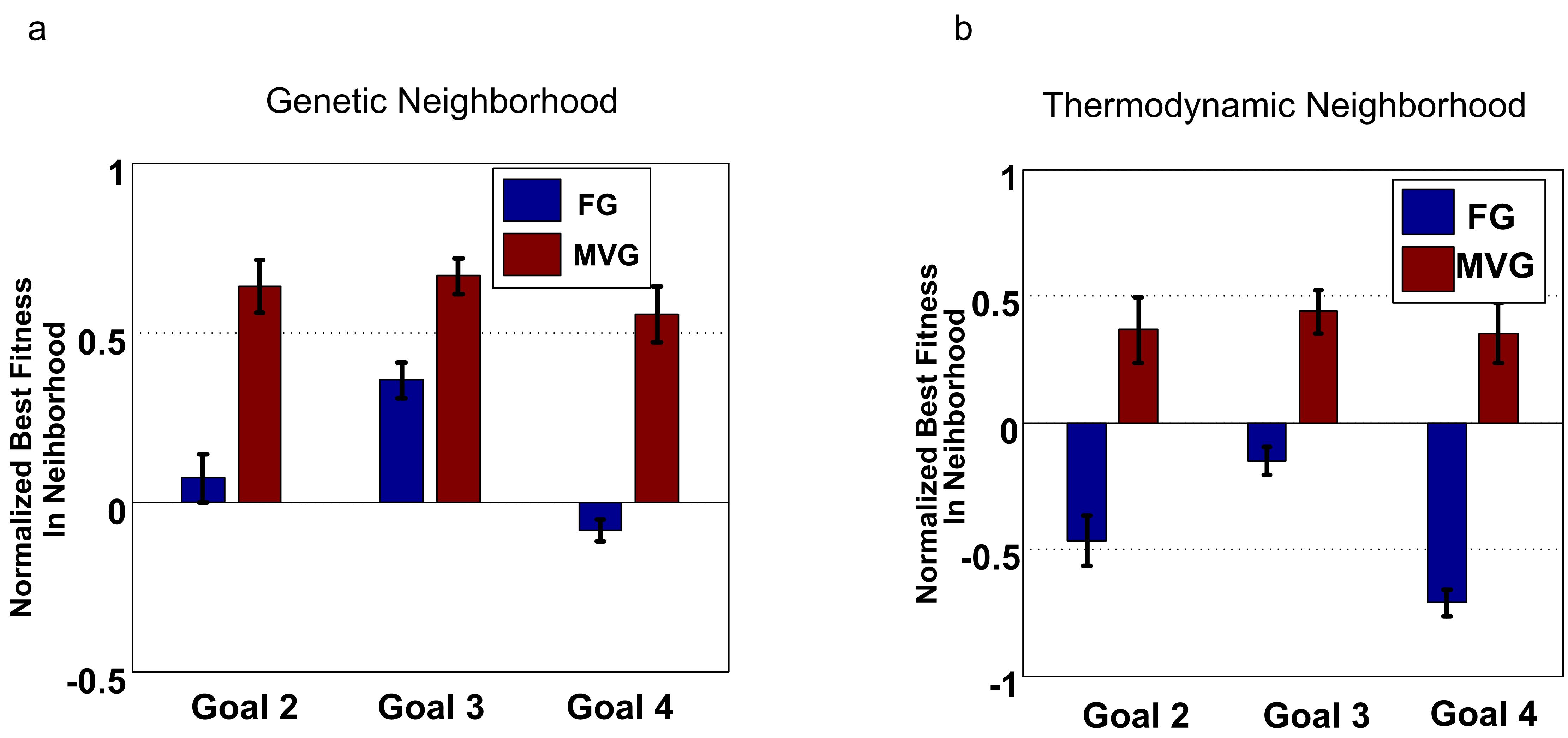
**

**Figure S10.** Plastogenic congruence [4] in RNA model. Maximal normalized fitness (mean ± SE) for past goals (G≠G1) in the **(a)** genetic neighboring structures **(b)** thermodynamic neighboring structures. Best 10 genomes from the end of the last G1-epoch population were analyzed. Data are from 30 simulations for each scenario,

**6.**  **Adaptation toward novel goals**

**6.1 Classes of novel goals**

We define three classes of novel goals. Two classes within MVG-context:

- *New-comb* goals
- *Novel-module* goals

And a third class which is not related to MVG history

- *Random novel* goals

For all three classes, trivial functions (such as "always zeros/ones" Boolean functions) were avoided.

**6.2 Controlling complexity of novel goals**

The complexity of a goal can be estimated by the median time to find solutions for the goal, from an initial random population under fixed goal environment. However, measuring complexity in this manner is not practical, if one analyzed a large set of goals.

Therefore, we sought for a correlative measure for goal's complexity. To address this, we mapped the entire genotypic space of a smaller model of logic circuits (which allows various gate types and not only NAND gates), described in Ref [2, 3]. The genome in this model was only of 38 bits, thus it was feasible to map all 2^38^~10^11^ genotypes to their corresponding phenotypes (Boolean functions). We then used the number of solutions of a given Boolean function as an estimate for its hardness. For the adaptation analysis (with the three classes of novel goals), we considered goals with less than 10^6^ solutions. For MVG language definition, only decomposable goals with less then 10^8^ solutions were considered.

**6.3 Methods for comparing populations’ evolvability**

We applied two procedures in order to compare between FG and MVG evolvability^*1^:

**6.3.1** Populations were evolved toward a previously un-seen goal for 500 generations. Fig. S11a indicates that MVG out-performance on FG populations when presented to *new-comb* goal in logic circuit model.

**6.3.2** MVG and FG were competing for survival toward new goals in the following manner, *1*. The starting population was composed of 50% of the individuals taken from FG-organisms and 50% MVG-organisms. *2*. The mixed MVG-FG population was evolved under new goal for *L*^*2^=40 generations. Since population size was kept constant, MVG and FG were competing for space. *3*. The survivors were traced for their FG/MVG ancestors in the starting population. *4*. The winner (MVG/FG) was the one with more surviving decedents.

Figure S11b presents the result of such a competition for a *new-comb* goal (logic circuit model).

Comments:

^*1^ Only populations that included at least one solution to G1 were analyzed.

^*2^  By that time, most of the population is originated from a single common ancestor.

**Figure S11**

**
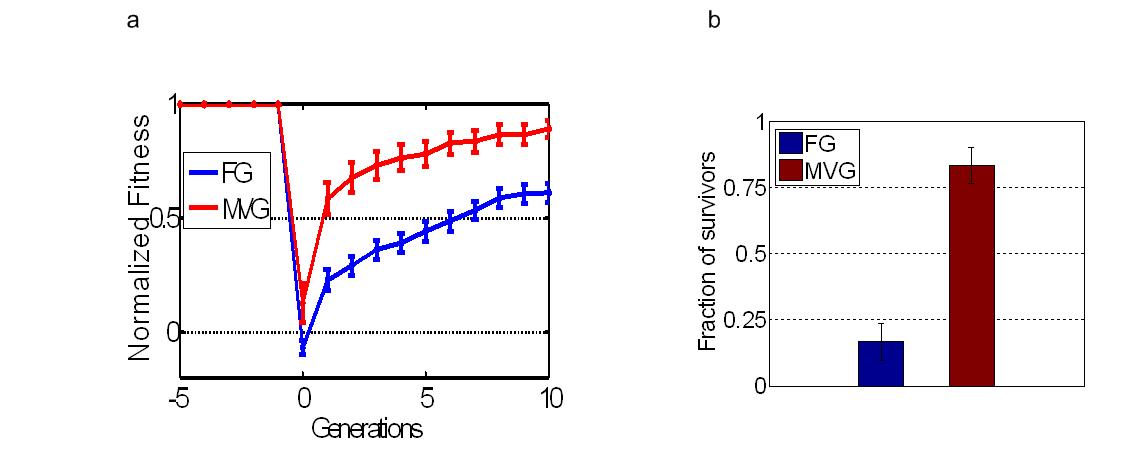
**

**Figure S11.** Comparing FG and MVG evolvability toward *new-comb* goals (logic circuit model). MVG evolution was as described in Fig. 2A. The *new-comb* goal is G_comb_=(x EQ y) OR (w EQ z). **(a)** Maximal normalized fitness in the logic circuits populations (mean ± SE) as a function of generations for G_comb_ goal. The initial populations were: FG-populations evolved toward G1, MVG populations taken from the end of the last G1-epoch. Data are averaged for 30 simulations in each case. **(b)** Competition results.

**6.4 Adaptation toward *novel-module* goals**

**6.4.1 The relation between speedup and goal’s complexity** **(model-1)**

We analyzed MVG and FG adaptation toward *novel-module* goals with 4 thresholds of complexity (following section 6.2). We find that the harder the novel-module goal is, the more pronounced is the speedup of MVG populations.

**Figure S12**

**
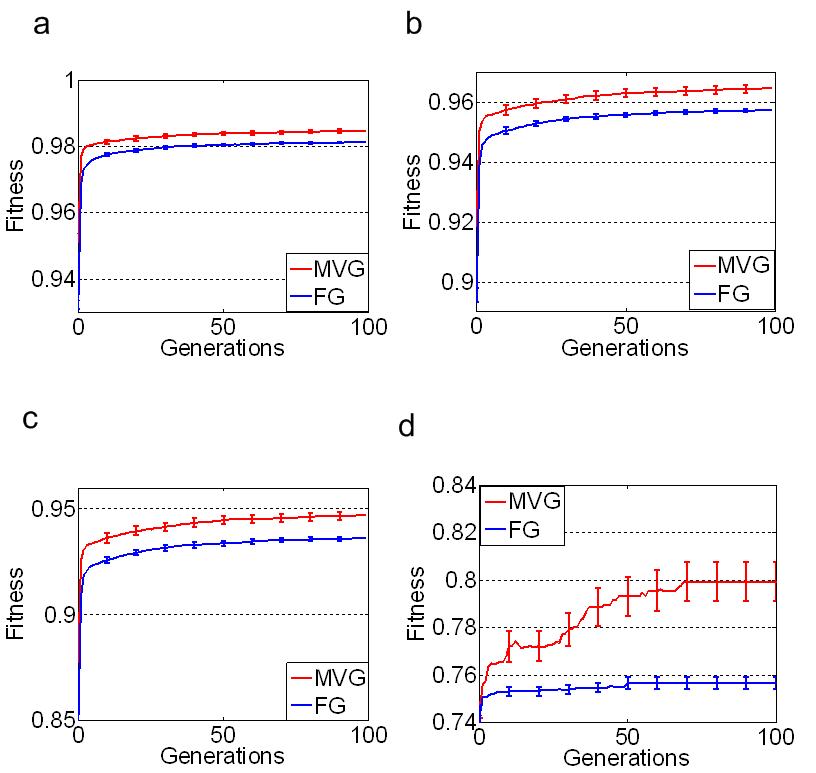
**

**Figure S12.** Comparing FG and MVG evolvability toward *novel-module* goals with increasing levels of complexity. MVG goals were G1=(x XOR y) AND (w XOR Z) , G2=(x **EQ** y) AND (w XOR Z) and G3 = (x XOR y) AND (w **EQ** Z). Shown is maximal fitness in the population (mean ± SE) as a function of generations. **(a)** Goals with less than 10^8^ solutions, such as G=(x XOR y) AND (w **NOR** Z). **(b)** Goals with less than 10^6^ solutions, for example, G = (x **AND not** y) AND (w XOR z). **(c)** Goals with less than 10^5^ solutions, such as G=(x XOR y) AND (w **🡪** z). **(d)** Goals with less than 10^4^ solutions, such as G = (x XOR y) **XOR** (w XOR z). For MVG, the ends of the last G1-epoch populations are taken as the initial populations. Data are from 30 simulations (in each scenario).

**6.4.2 List of novel-module goals studied**

For the main example of Fig. 2A where G1= (x XOR y) OR (w XOR z), the novel-module goals studied are listed below. Those goals correspond to goals with less than 10^6^ solutions

(following section 6.2), and that were not introduced in MVG evolution (i.e. novel goals for both FG and MVG evolved populations) .

The right hand side corresponds to the truth table of the Boolean function; the "novel" module is in bold.

(1) (x XOR y) **AND** (w XOR z) = 0660

(2) (x XOR y) **XOR** (w XOR z) = 6996

(3) (x XOR y) **NAND** (w XOR z) = F99F

(4) (x XOR y) **EQ** (w XOR z) = 9669

(5) **(x AND y)** OR (w XOR z) = 666F

(6)  **(x AND n y**) OR (w XOR z) = 66F6

(7)  **(x)** OR (w XOR z) = 66FF

(8)  **(n x AND y)** OR (w XOR z) = 6F66

(9)  **(y)** OR (w XOR z) = 6F6F

(10) **(y 🡪 x)** OR (w XOR z ) = F6FF

(11) **(x 🡪 y)** OR (w XOR z) = FF6F

(12) **(x NAND y)** OR (w XOR z) = FFF6

(13) (x XOR y) OR **(w AND z)** = 1FF1

(14) (x XOR y) OR **(w AND n z)** = 2FF2

(15) (x XOR y) OR **(w)** = 3FF3

(16) (x XOR y) OR **(n w AND z)** = 4FF4

(17) (x XOR y) OR **(z)** = 5FF5

(18) (x XOR y) OR **(z 🡪 w)** = BFFB

(19) (x XOR y) OR **(w 🡪 z)** = DFFD

(20) (x XOR y) OR **(w NAND z)** = EFFE

**6.5 Adaptation toward *random novel* goals**

**Figure S13**

**
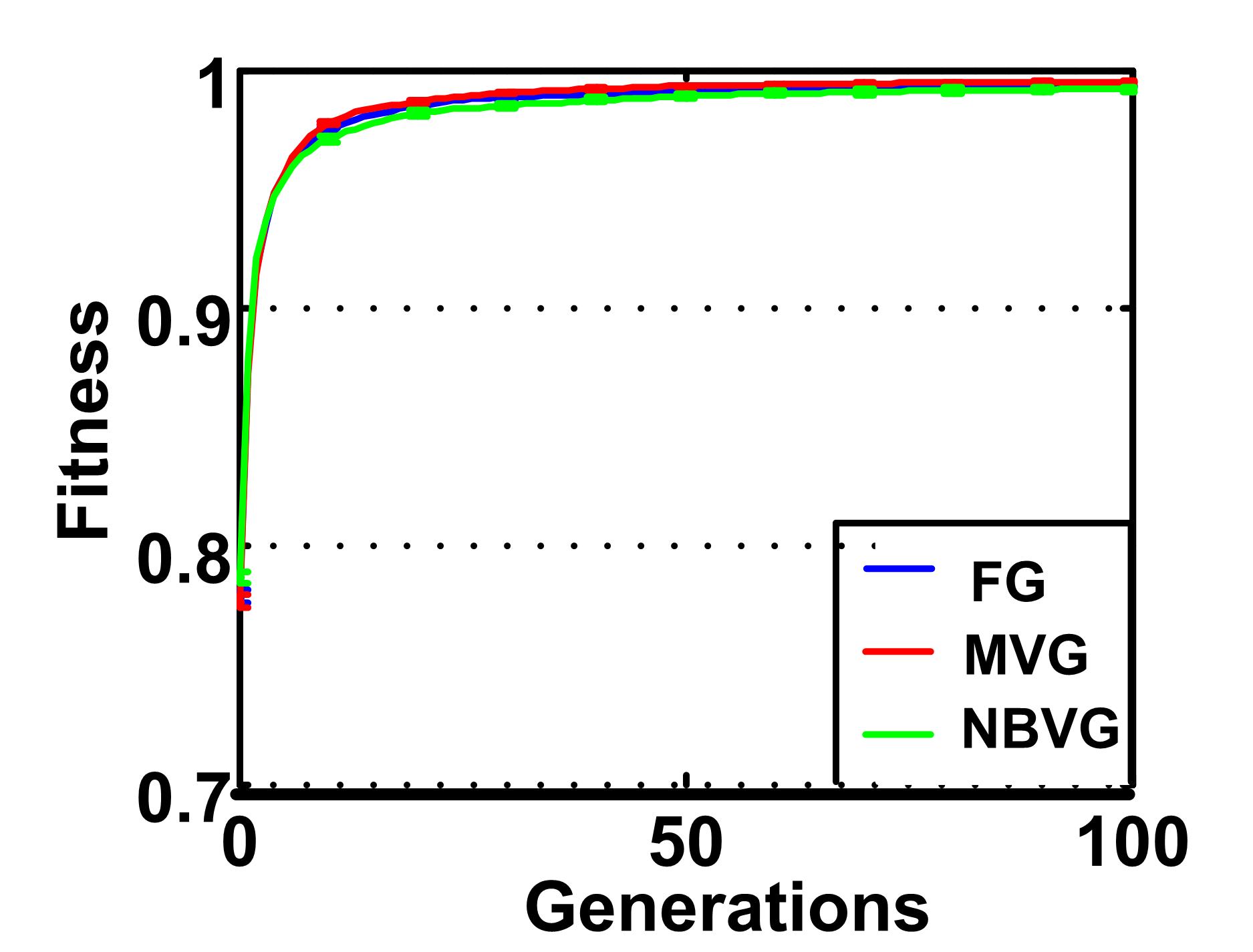
**

**Figure S13.** Adaptation toward *random novel* goals with three scenarios of evolution (logic circuit model).

MVG evolution was as described in Fig. 2A, NBVG evolution is as described in section 2.1. The end of the last G1-epoch population is taken as the initial population. Data are from 30 simulations (in each scenario) and for 50 *random novel* goals.

**Random Goals in RNA model**

A random set of RNA secondary structures was constructed by folding 1000 random RNA sequences. In principle, the complexity of a random goal (structure) could be controlled by mapping many random genotypes, and then considering only phenotypes with a unique appearance. However since genome length is ~70 bases, even unique structures in a sample of 10^7^ genomes can still have large neutral networks (<=4^70^/10^7^).

We find that MVG populations are as evolvable as FG populations toward random structures whose appearance was unique in a sample of 10^7^ genomes.

**7. Complete characterization of evolved phenotypic neighborhoods (model-2)**

We find that the neighborhood of MVG-circuits is enriched (relative to FG) with circuits that compute decomposable Boolean functions, i.e. functions of the form *u*(x,y,z,w)=*f*(g(x,y),*h*(z,w)) (Fig. S14a). The class of non-MVG phenotypes includes trivial functions and non-trivial functions that are not decomposable (according to the latter definition). We also find that FG neighboring circuits are significantly less modular (Fig. S14b).

**Figure S14**

**
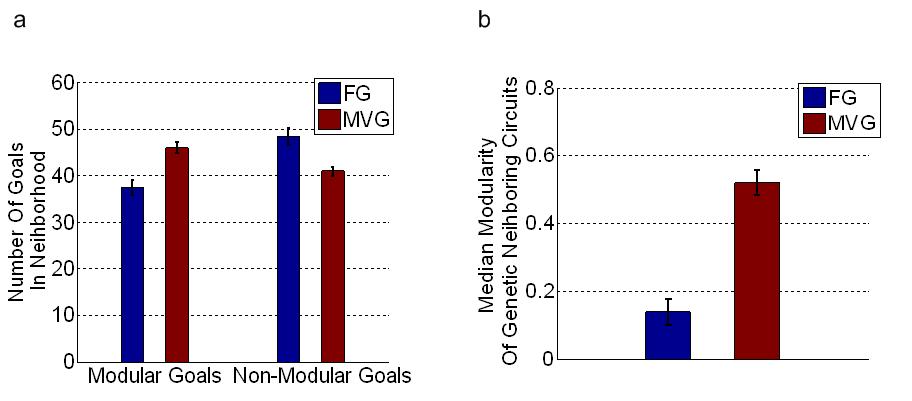
**

**8. Facilitated variation analysis**

**8.1 Facilitated variation measure**

The suggested FV measure (see main-text) is a product of quality and quantity components. The quality component corresponds to the likelihood of the individual to generate a potentially useful phenotype upon one genetic mutation. The quality component further corresponds to two aspects of a desired variation: (a) large phenotypic diversity of useful novel phenotypes and (b) large phenotypic distance between novel useful phenotypes and the wild-type. The first is evaluated by measuring the average variation among all potential useful phenotypes in the phenotypic neighborhood. The second is evaluated by measuring the average phenotypic distance between the wild-type and the potentially useful phenotypes within the phenotypic neighborhood. We find that these two measures are highly correlated in the two model systems (Fig. S15). Thus it is sufficient to include only one of these measures in FV measure.


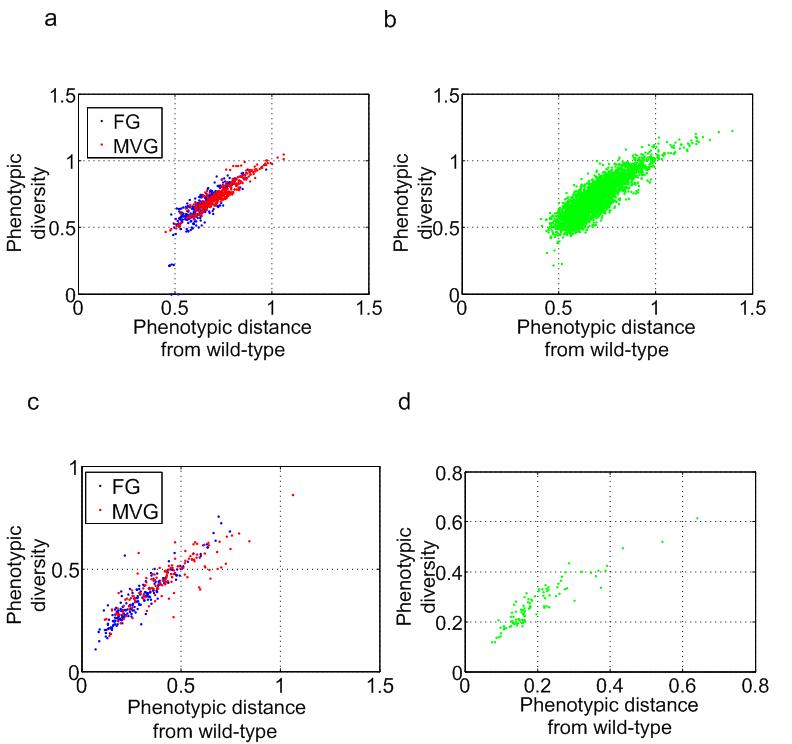
**Figure S15**

**Figure S15.** The relation between two quality measures. X-axis is the ratio between useful phenotypic diversity and non-useful phenotypic diversity (first measure). Y-axis is the ratio between the distance from wild-type to useful phenotypes and the distance from wild-type to non-useful phenotypes. **(a-b)** Logic circuit model, **(c-d)** RNA model. **(b)** and **(d)** shown are results for random samples of genomes obtained by **(b)** simulated annealing algorithm and by **(d)** inverse-fold algorithm.

**8.2 Evolution of facilitated variation on the neutral network**

An initial population of genomes was created by sampling *N*_pop_ = 5000 genomes from G_1_ = (x XOR y) AND (w XOR z) neutral-network (by means of simulated annealing algorithm).We evolved this population for *L*=5$\times$10^3^ generation, under three scenarios: MVG, FG and NBVG.

Whereas NBVG evolution (as described in section 2.1) did not affect FV, both FG and MVG environments, have enhanced FV along evolution. Importantly, FV enhancement is significantly larger in the case of MVG evolution (Fig. S16a). We also note that the increase in FV was followed by corresponding increase in Qm (Fig. S16b) and in mutational modularity measure (see section S9 and Fig. S16c).

**Figure S16**

**
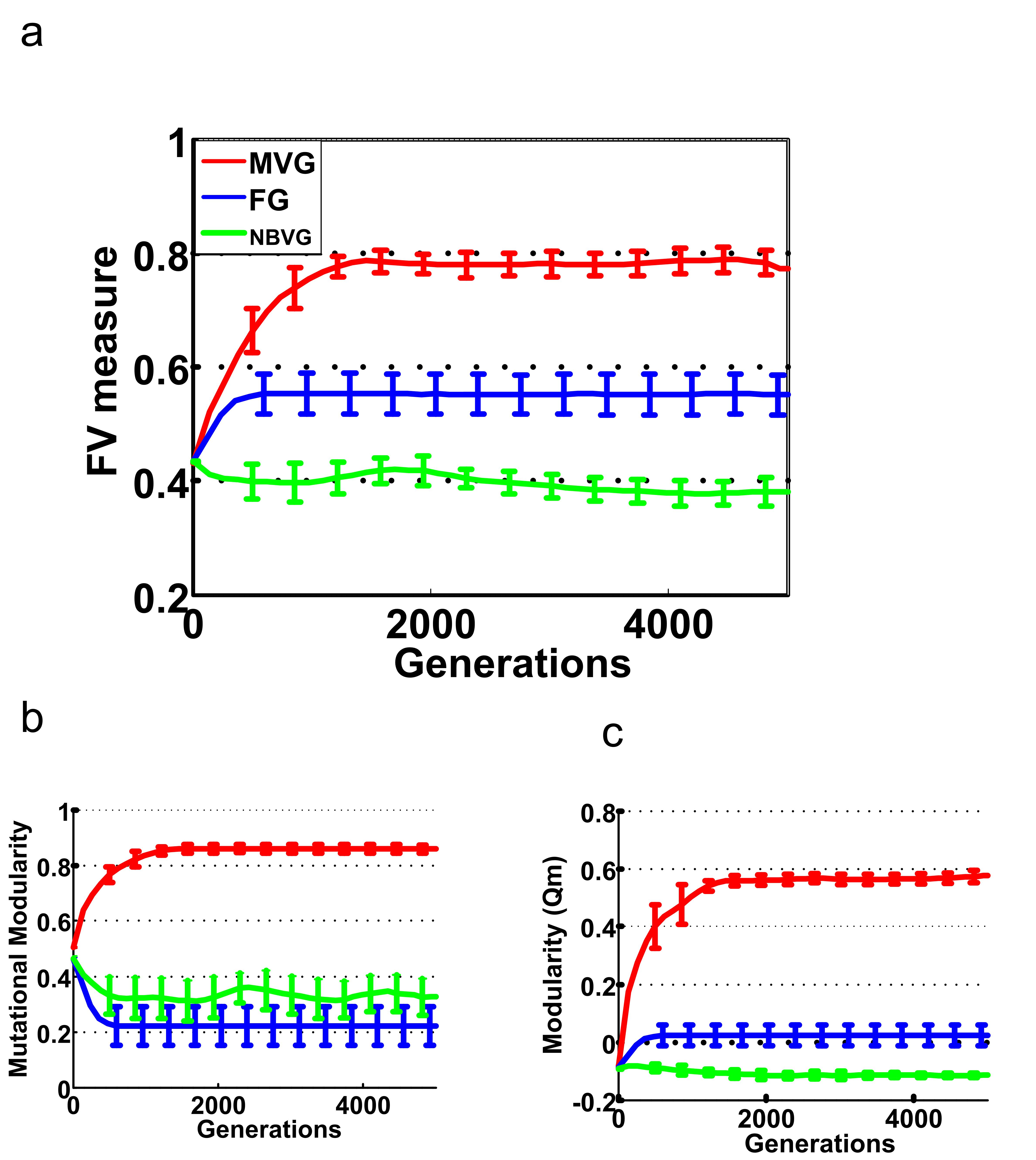
**

**Figure S16**. Dynamics of FV related measures on a neutral network. The initial population is a collection of *N*_pop_ solutions obtained by SA (simulated annealing) algorithm. This initial population was further evolved for *L*=5$\times$10^3^ generations with MVG (red), FG (blue) and NBVG (green) scenarios. **(a)** FV measure **(b)** Mutational modularity measure (see next section) and **(c)** Structural modularity measure as a function of generations (x-axis). Each measure was averaged on 500 best individuals in the population. Data are for 30 simulations under each scenario.

**9. Quantitative measure of mutational modularity in logic circuit model**

To evaluate the modularity of the effects of mutations, we first computed a complete, directed, and weighted network of mutation effects. Each node in the network is a gene (a NAND gate) and a directed edge E_i,j_ corresponds to the phenotypic change in gene i upon genetic mutation in gene j. We then computed the Q measure of this network, using the division into modules of the original circuit. High Q values indicate that mutations have strong effects within their own module and weak effects on other modules (low pleiotropy). We find that the mutational-modularity measure as well as Qm are significantly more enhanced along MVG evolution (Fig. S17).

**Figure S17**

**
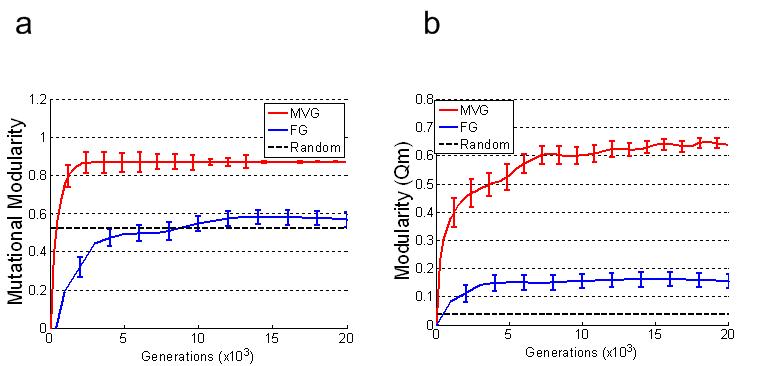
**

**Figure S17. (a)** Mutational modularity and **(b**) structural modularity (Q_m_) as a function of generations in logic circuits evolution, MVG goals are G1 = (x XOR y) **OR** (w XOR z), G2 = (x XOR y) **AND** (w XOR z).

**10. Conditional entropy measure**

**10.1 How to measure conditional entropy from population genomes?**

Consider a simple MVG scenario (model-2) that switches with equal rates between 4 goals: T1-T4 according to the transition network described in Fig. 2a. The probability to be at goal T1 is 1/2, and at each of the other goals the probability is 1/6. The probabilities of the genomic variable X given that the current goal is Ti are computed from the population genomes. The conditional genomic entropy in this case is then:

$$H(X|T)=\frac{1}{2}H(X|T=T_{1})+\frac{1}{6}H(X|T=T_{2})+\frac{1}{6}H(X|T=T_{3})+\frac{1}{6}H(X|T=T_{4})$$

**10.2 Genetic variance for large number of varying goals**

We find that when the number of varying goals introduced along evolution is above a certain threshold, the genetic variance of the population start to increase. The reason for that might be that for a large number of goals, there is no set of solutions to all goals that are in close genomic proximity.

**Figure S18**

**
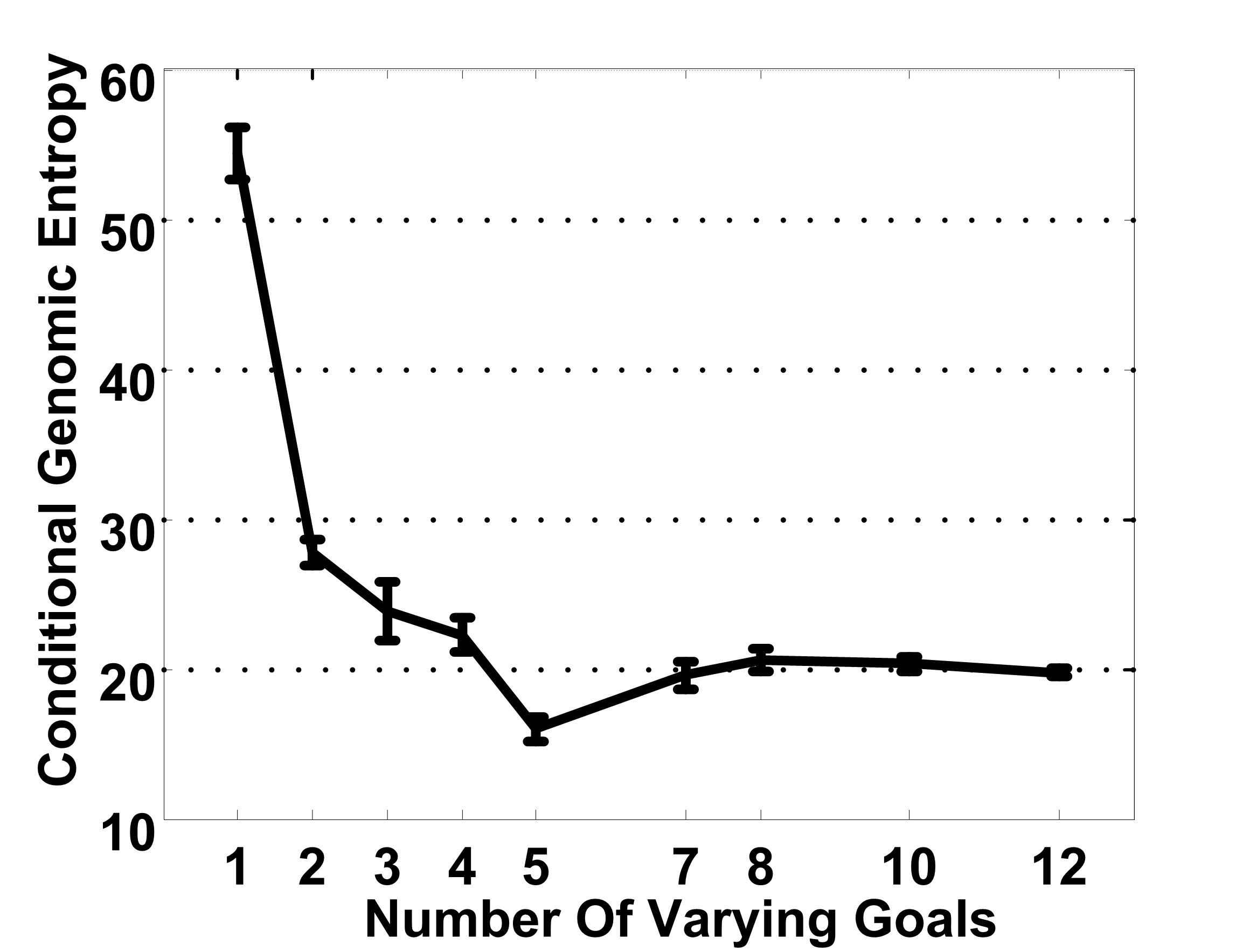
**

**Figure S18.** Conditional genomic entropy (Mean ± SE) as a function of number of goals presented along MVG evolution (x-axis).

**11. Characterization of evolved genome positions**

Inspired by Kirschner and Gerhart theory, we distinguish between three classes of genomic positions (Fig. S19a):

1. *Conserved* positions: positions that maintain their genomic content for many generations, and have identical content across multiple species (similarly to the ribosomal machinery).

2. *Trigger* positions: positions that are mutated with high probability in response to environmental change (between epochs), but have low chance of changing during epochs (i.e. when goal is constant), similarly to allosteric proteins.

3. *Random drift* positions: positions that have a high constant mutation rate which is non-correlated to the environment (with analogy to "junk" DNA regions).

Two of the above position classes have no correlation to the environment: the conserved position that have a constant low rate of changing, and the random drift positions that have constant high rate of changing. The triggers are the positions with a non-constant rate of change; their rate is highly correlated to the changing rate of the environment (goal).

**11.1 conserved positions**

We detected the conserved positions in the genome by computing the marginal entropy of each site. The conserved positions correspond to the lowest marginal entropy positions.

The marginal entropy at site X_i_  is computed in the following manner:

$\alpha=P(X_{i}=j|T=T_{k}),j=\{A,U,G,C\},K=\{1,2,3,4\}$ [1]

$\beta=P(X_{i}=j)=\sum_{K=\{1,2,3,4\}} \alpha P(T=T_{k})$ [2]

$H(X_{i})=-\sum_{j=\{A,U,G,C\}} \beta\log(\beta)$ [3]

The notations are as described in section 10. Note that in FG evolution, the conditional genomic entropy is equivalent to the marginal entropy (since the entropy in the environment is zero) thus one can compute the marginal entropy simply from the probabilities obtained from the population.

We also suggest another procedure for detecting conserved positions. The proposed alternative aims to mimic the commonly applied biological method. The conventional method for detecting such positions is to analyze genomes of distantly related species and to find common sub-sequences. We followed this logic, by creating distant populations (species) from a common ancestor population, by means of distinct evolutionary simulations starting form the same initial population. We then measured the time that a mutation first occurred in each position averaged over the number of species we evolved. Based on this, a conservation score was given to each position defined as the probability to maintain the genomic content.

We find good agreement between two methods described (Fig. S19b).

**11.2 Random-drift positions**

In order to detect the random-drift positions we measured the conditional genomic entropy as described in section 11.1. Random drift positions have high rate of changing which is not correlated to environment. Therefore those positions are characterized with high conditional genomic entropy. Note that random drift positions are also characterized with high marginal entropy, and yet high marginal entropy is not a *characteristic* feature since it may be common for both random drift and triggers positions (in case the environment changes in high rate).

Therefore, the key feature for distinguishing between a genetic trigger position to a random-drift one is the conditional entropy of that site: whereas triggers are constrained in non-changing periods along evolution, the random-drift are changing in a constant rate implying high conditional entropy even when the goal is constant .

**11.3 Genetic triggers**

The genetic triggers were identified by measuring the mutual information between the environment (goal) and the genomic content.


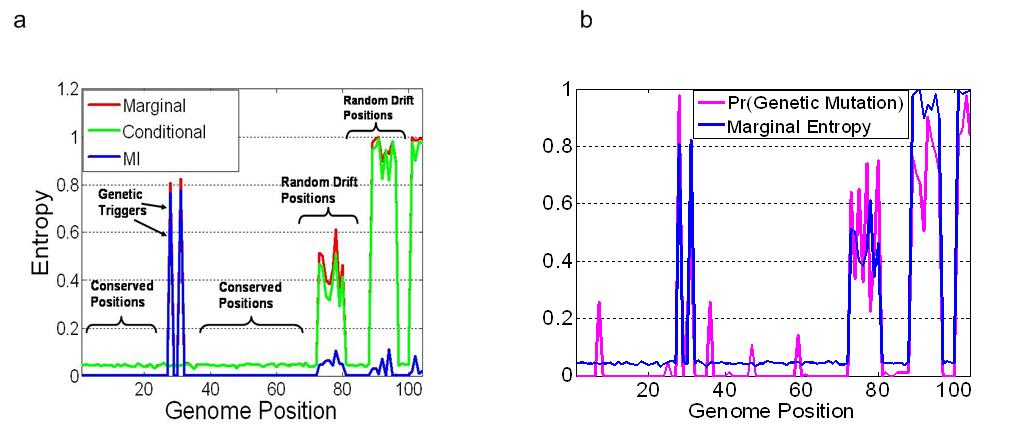
**Figure S19**

**Figure S19.** **(a)** Evolution of trigger, conserved and drift genomic positions. Shown are the marginal entropy (red), conditional entropy (green) and mutual-information (MI) as a function of genome positions (logic circuit model). **(b)** Detection of conserved positions on evolved genome. Marginal entropy (blue) and conservation score of distantly related genomes (magenta) are shown as a function of genomic positions.

**References**

1. Mitchell M (1996) An Introduction to Genetic Algorithms. Cambridge MA: MIT Press.

2. Kashtan N, Alon U (2005) Spontaneous evolution of modularity and network motifs. Proc Natl Acad Sci U S A 102: 13773-13778.

3. Kashtan N, Noor E, Alon U (2007) Varying environments can speed up evolution. Proc Natl Acad Sci U S A 104: 13711-13716.

4. Ancel LW, Fontana W (2000) Plasticity, evolvability, and modularity in RNA. J Exp Zool

288: 242-283.

5. Newman MEJ, Barkema GT (1999) Monte Carlo Methods in Statistical Physics: Oxford University Press.

6. Hofacker IL (2003) Vienna RNA secondary structure server. Nucleic Acids Res 31: 3429-3431.

7. Borenstein E, Ruppin E (2006) Direct evolution of genetic robustness in microRNA. Proc Natl Acad Sci U S A 103: 6593-6598.
